# Supplementary material for: Factors associated with COVID-19 vaccine uptake among people with type 2 diabetes in Kenya and Tanzania: a mixed-methods study
Source: BMJ Open. 2023 Dec 7;13(12):e073668. doi: 10.1136/bmjopen-2023-073668 (PMC10711896; doi:10.1136/bmjopen-2023-073668)
Supplement: Supplementary data [file bmjopen-2023-073668supp001.pdf]

# Appendix

**Appendix Table 1: Multivariate Modified Poisson regression results of potential predictors of COVID-19 vaccine uptake**

| Variable | Description | Kenya | | Tanzania | |
|---|---|---|---|---|---|
| | | RR (p-value) | (95%CI) | RR (p-value) | (95%CI) |
| **Place of residence** | Urban (ref) | 1.00 | | 1.00 | |
| | Rural | 1.01 (0.900) | (0.90-1.12) | 0.90 (0.554) | (0.63-1.29) |
| **Sex** | Female (ref) | 1.00 | | 1.00 | |
| | Male | 1.02 (0.751) | (0.91-1.14) | 1.17 (0.355) | (0.84-1.63) |
| **Marital status** | Not married (ref) | 1.00 | | 1.00 | |
| | Married | 1.10 (0.118) | (0.98-1.25) | 1.37 (0.085) | (0.96-1.97) |
| **Age group** | <40 years (ref) | 1.00 | | 1.00 | |
| | 40-49 years | 1.15 (0.411) | (0.82-1.62) | 1.16 (0.760) | (0.45-3.00) |
| | 50-59 years | **1.33 (0.071)** | **(0.98-1.82)** | 1.38 (0.463) | (0.59-3.24) |
| | 60-69 years | **1.33 (0.071)** | **(0.98-1.83)** | 1.72 (0.214) | (0.73-4.03) |
| | > 70 years | **1.33 (0.085)** | **(0.96-1.85)** | 1.74 (0.249) | (0.68-4.44) |
| **Education level** | No education (ref) | 1.00 | | 1.00 | |
| | Primary education | 1.07 (0.690) | (0.78-1.46) | 1.41 (0.481) | (0.55-3.62) |
| | Secondary education | 1.09 (0.583) | (0 80-1.50) | 1.63 (0.327) | (0.61-4.36) |
| | Higher education | 1.27 (0.144) | (0.92-1.77) | **2.63 (0.065)** | **(0.94-7.35)** |

| | | | | | |
|---|---|---|---|---|---|
| **Religion** | Catholic (ref) | 1.00 | | 1.00 | |
| | Protestants | 0.97 (0.611) | (0.87-1.09) | 1.11 (0.581) | (0.76-1.61) |
| | Muslims | 0.87 (0.448) | (0.61-1.25) | 0.95 (0.770) | (0.67-1.35) |
| **Occupation status** | Formal workers (ref) | 1.00 | | 1.00 | |
| | Farmers | 1.18 (0.137) | (0.95-1.46) | 1.08 (0.809) | (0.57-2.07) |
| | Self-employed business | 1.05 (0.631) | (0.87-1.26) | 1.10 (0.765) | (0.58-2.08) |
| | Taking care of home | 0.98 (0.900) | (0.69-1.39) | | |
| | Retired | 1.03 (0.825) | (0.82-1.28) | 0.93 (0.819) | (0.49-1.75) |
| | Unemployed | 0.91 (0.384) | (0.73-1.13) | 0.98 (0.949) | (0.46-2.07) |
| **Health insurance** | Insured | 1.14 (0.035) | (1.01-1.29) | 1.56 (0.045) | (1.01-2.41) |
| | Not insured (ref) | 1.00 | | 1.00 | |
| **Socioeconomic status (ladder scale 1-10)** | Lower SES (1–5) | 0.96 (0.637) | (0.82-1.13) | 0.74 (0.075) | (0.53-1.03) |
| | Higher SES (6–10) (ref) | 1.00 | | 1.00 | |
| **Family history of T2D** | Yes | 1.08 (0.130) | (0.98-1.19) | 1.22 (0.189) | (0.91-1.65) |
| | No (reference) | 1.00 | | 1.00 | |
| **Time living with T2D** | <6 years | 0.93 (0.208) | (0.84-1.04) | 0.82 (0.266) | (0.57-1.17) |
| | ≥6 years (ref) | 1.00 | | 1.00 | |
| **Comorbidities** | Number of comorbidities | 1.02 (0.629) | (0.95-1.10) | 1.18 (0.022) | (1.02-1.35) |
