# Supplementary material for: Factors associated with COVID-19 vaccine uptake among people with type 2 diabetes in Kenya and Tanzania: a mixed-methods study
Source: BMJ Open. 2023 Dec 7;13(12):e073668. doi: 10.1136/bmjopen-2023-073668 (PMC10711896; doi:10.1136/bmjopen-2023-073668)
Supplement: Supplementary data [file bmjopen-2023-073668supp002.pdf]

ISO 9001: 2015 certified
IFAKARA HEALTH INSTITUTE
research | training | services

APHRC African Population and Health Research Center

Healthcare and Socio-economic Impacts of COVID-19 on Patients with type 2 Diabetes in Kenya & Tanzania
Athari za Kiafya na Kiuchumi za UVIKO-19 kwa Wagonjwa wa Kisukari cha Aina ya 2 nchini Kenya na Tanzania

Indepth Interview guide for individuals with type 2 diabets
Mwongozo wa Mahojiano ya Kina kwa Wagonjwa wa Kisukari cha Aina ya 2

Before we start the interview, I would like us to read the informed consent form together, and then you can let me know if you want to be interviewed.

Kabla hatujaanza mahojiano, ningependa tusome pamoja fomu ya ridha, kisha unaweza kunijulisha ikiwa unapenda kuhojiwa.

Research assistant: (remember to collect the social demographic characteristics of the participants before starting the interview)

Msaidizi wa utafiti: *(kumbuka kukusanya sifa za idadi ya watu kijamii ya washiriki kabla ya kuanza mahojiano)*

**DEMOGRAPHIC INFORMATION**<br>**TAARIFA ZA KIDEMOGRAFIA**	
**Respondent name/code**<br>**Jina la mhojiwa/msimbo**	
**Age**<br>**Umri**	
**Education**<br>**Elimu**	
**Marital status**<br>**Hali ya ndoa**	
**Economic income/position**<br>**Mapato ya kiuchumi/nafasi**	
**Religion**<br>**Dini**	

**Occupation**<br>**Kazi**	
**Tribe**<br>**Kabila**	
**Village**<br>**Kijiji**	
**District**<br>**Wilaya**	
**Region**<br>**Mkoa**	
**Contact**<br>**Mawasiliano**	

Section A: Illness history

Sehemu A: Historia ya Ugonjwa

Please tell me more about your experience with diabetes condition since you were diagnosed with diabetes[FI *Instruction: Ask the respondent to think back to the time from when you were diagnosed]*

Tafadhali niambie uzoefu wako kuhusiana na ugonjwa wa kisukari tangu ulipogunduliwa na ugonjwa wa kisukari *[Maelekezo ya FI: Mwambie mhojiwa afikirie kipindi tangu alipogundua kuwa na ugonjwa wa kisukari]*

Dodosa:

a) What symptoms,if any,did you experience?
Je, ulikuwa na dalili gani?

b) How did you feel about being diagnosed with diabetes?
Ulijisikiaje ulipogundulika kuwa na ugonjwa wa kisukari?

c) How did your family, friends and community members respond to you having diabetes?
Je familia yako,marafiki na wanajamii waliopokea vipi suala la ugonjwa wako wa kisukari?

1. **Do you have any other health conditions apart from diabetes?**
Je, una shida nyingine ya kiafya mbali na ugonjwa wa kisukari?
If the response is yes, probe the following
Ikiwa jibu ni ndiyo, dodosa yafuatayo:

a) Please briefly talk about your experience living with diabetes ?
Tafadhali nieleze kwa ufupi ni jinsi gani umekuwa ukiishi na ugonjwa huu wa kisukari?

b) Has it been easy or difficult for you to live with all these health problems? explain more
Je imekuwa rahisi au ngumu kwako kuishi na shida zote hizi za kiafya? eleza zaidi

c) Has this condition affected how you control your diabetes?
Je, hali hii imeathiri jinsi unavyodhibiti hali yako ya ugonjwa wa kisukari ?

Section B: Patient experience in illness management before COVID-19

Sehemu B: Uzoefu wa mgonjwa katika udhibiti wa magonjwa kabla ya UVIKO-19

In this section we are going to discuss questions about how you have **MANAGE** your diabetes and access to diabetes care services **BEFORE** the COVID-19 period, that is before March 2020

Katika sehemu hii tutajadili maswali kuhusu namna ambavyo umeweza **KUDHIBITI** ugonjwa wako wa kisukari na upatikanaji wa huduma za matibabu ya kisukari **KABLA** ya kipindi cha UVIKO-19, yani kabla ya Machi 2020

2. **There are different ways of managing diabetes, please tell me how you were managing your diabetes in the period before COVID-19 (*do not forget asking about modern medical ways such as taking medication, injections, exercise, diet and using natural remedies*)**

   Kuna njia mbalimbali za kudhibiti ugonjwa wa kisukari, tafadhali niambie jinsi ulivyokuwa ukidhibiti ugonjwa wako wa kisukari katika kipindi cha kabla ya UVIKO-19 *(usiache kudadisi kuhusu njia za kitabibu za kisasa kama kunywa dawa, sindano, mazoezi, chakula na kutumia njia za asili)*

3. **Thinking about how you have been managing diabetes** (*provide an example of the activity that the patient mentioned above)* **how has your management of diabetes changed over time (here we are still referring to the period before COVID-19)?**

   Fikiria namna ambavyo umekuwa ukidhibiti ugonjwa wa kisukari*(toa mfano wa shughuli ambayo mgonjwa alitaja hapo juu)* **je udhibiti wako wa ugongwa wa kisukari umekuwa ukibadilika? (hapa bado tunazungumzia kipindi cha kabla ya UVIKO-19)?**

   Probe: What has changed? Please tell us more
   Dodosa: Nini kimebadilika? Tafadhali tuelezea zaidi.

4. **What type of assistance have you been receiving in managing your diabetes? (*remind the participant that you are reffering to the period before COVID-19*)**

Umekuwa ukipokea msaada wa aina gani katika kudhibiti ugonjwa wako wa kisukari? (mkumbushe mshiriki kuwa unarejea kipindi cha kabla ya UVIKO-19)

Probe: Did anyone help you to take care of your diabetes? tell us more

Dodosa: Je, kuna mtu yeyote aliyekusaidia kukuhudumia katika ugonjwa wako kisukari?

Tell us more:Family member, health care provider, traditional healers?

Tuambie Zaidi (uliza kuhusu: mwanafamilia, mhudumu wa afya, waganga wa kienyeji?)

Probe: Was the assistance helpful? why?

Dodosa: Je, msaada huo ulisaidia? kwa nini?

We have spoken about how you manage your diabetes. Now lets talk about how you **ACCESS** diabetes health care services in the period **BEFORE** COVID-19.

Tumezungumza juu ya namna unavyo **DHIBITI** ugonjwa wako wa kisukari. Sasa hebu tuzungumze kuhusu **UFIKIAJI** wako wa huduma za matibabu ya ugonjwa wa kisukari katika kipindi cha kabla ya UVIKO-19

5. **Tell us about where you sought diabetes health care services?**
   Tuambie unapata wapi huduma za matibabu ya ugonjwa wa kisukari?

Ddosa:

a) Probe: what type of services did you receive from the health facility?
   Je ni aina gani ya huduma ulizopokea kutoka kwa kituo cha afya?
b) Probe: how helpful were the services that you received? Tell us more
   Je huduma ulizopokea zilikua na manufaa kiasi gani? Tuambie Zaidi
c) Probe: how did the services met your expectations? Why?
   Je ni kwa namna gani huduma za kiafya zilikidhi vipi matarajio yako? Kwa nini?

6. **What information did you get about controlling your diabetes?**
   Dodosa: Ni taarifa zipi umepata juu ya kudhibiti ugonjwa wako wa kisukari?

a) Probe: who provided that information?
   Nani alikupa taarifa hizo?
b) Probe: was the information helpful? how?
   Je taarifa hizi zilikuwa na msaada? Ki vipi?

*Think about things that might have made accessing diabetes care services easy or difficult in the period before COVID-19 (**before March** 2020)*

*Fikiri kuhusu mambo ambayo huenda yalifanya upatikanaji wa huduma za matibabu ya kisukari kuwa rahisi au ngumu katika kipindi cha kabla ya UVIKO-19 (**kabla ya Machi** 2020)*

7. **How difficult/easy was it for you to access diabetes services in the period before COVID-19? (why)**
   Je, ilikuwa vigumu/rahisi vipi kwako kupata huduma za matibabu ya ugonjwa wa kisukari kabla ya UVIKO-19? (kwanini?)

Dodosa:

a) Probe: what was the difficult/easy aspect?
   Ni kitu gani hasa kilikuwa gumu/rahisi kwako?

b) Probe: how did you handle this difficult?
   Je ni kwa namna gani uliweza kukabiliana na ugumu huo?

c) Was it easy for you to handle this difficult?
   Je ilikuwa rahisi kwako kutatua hiyo hali ngumu/changamoto hiyo?
d) How did that challenge / situation affect your diabetes management?
   Je chagamoto/hali hiyo ngumu iliathiri vipi kusimamizi wako wa ugonjwa wa kisukari?

e) Probe: did you find help in managing this diffiuclt? Tell us more
   Je, ulipata msaada katika kudhibiti changamoto hiyo/hali hiyo ngumu? Tueleze Zaidi

Ask: Can you tell me exactly before COVID-19 you were experiencing any difficulty in the following aspects?

Uliza: Unaweza kuniambia haswa kabla ya UVIKO-19 ulikuwa unapata ugumu wowote katika vipengele vifuatavyo?

- Transport, cost, availability of the services – how?
- Usafiri, gharama za kwenda kwenye huduma za afya?
- Access to medication- how?
  Upatikanaji wa huduma - kivipi?
- Access to medicine - how?
  Upatikanaji wa dawa - vipi?
- See a doctor - How?
  Kumuona daktari-Kivipi?
- Adhering medical advice? - how?
- Kuzingatia ushauri wa matibabu? - kivipi?
- Distress, fear? What exactly were you thining ?
  Msongo wa mawazo, hofu? Ulikuwa unawaza nini hasa?
- Food? (how)
  Chakula? Kivipi?
- Getting councelling, how?
  Kupata ushauri, kivipi?
- Support from relatives,how?
  Msaada kutoka kwa ndugu, kivipi?

DURING COVID-19 PERIOD
WAKATI WA UVIKO-19

Section C: Patient experience in illness management since the beginning of the COVID-19 pandemic
Sehemu C: Uzoefu wa mgonjwa katika udhibiti wa ugonjwa tangu mwanzo wa janga la UVIKO-19

In this section we are going to talk about how you have **MANAGED** your diabetes, and how you have **ACCESSED** the diabetes care services **SINCE** COVID-19 (We mean the period after March 2020)

Katika sehemu hii tutazungumzia namna gani ambavyo ulikuwa **UKIDHIBITI** ugonjwa wako wa kisukari, na jinsi ulivyofikia huduma za **UDHIBITI**I wa ugonjwa wa kisukari **TANGU** UVIKO-19 kuanza (tunamaanisha kipindi baada ya Machi 2020)

8. **Please tell me what ways you have been using to control your diabetes during COVID-19? (After March 2020)**
   Tafadhali niambie njia gani ulikuwa ukitumia kudhibiti ugonjwa wako wa kisukari wakati wa UVIKO-19? (Baada ya Machi 2020)

   Probe: Did these ways of controlling diabetes helped you in COVID-19 period? If not, why? If helped, why do you think so?
   Probe:Probe: *Je njia hizi za udhibiti ugonjwa wa kisukari zimekusaidia wakati huu wa UVIKO-19? Kama hazikusaidia unafikiri kwa nini? Kama zilisaidia unafikiri kwa nini?*

9. **Are there any changes in how you were managing diabetes before COVID-19 and during COVID-19? (*give an example of what respondent mentioned above like; using medicine, nutrition, exercise, etc ask if it changed)***
   Je kuna mabadiliko yoyote katika kudhibiti ugonjwa wa Kisukari kabla ya UVIKO-19 na wakati wa UVIKO-19? *(toa mfano, jambo ambalo mgonjwa alitaja hapo juu kama vile: kumeza vidonge, lishe, mazoezi, uulize kama kilibadilika)*

   Probe: What has changed? Please tell me more
   Dodosa: Nini kimebadilika? Tafadhali nielezee Zaidi

   Probe: What do you think has contributed to these changes?
   Dodosa: Unafikiri nini kimechangia mabadiliko haya?

   Probe: Did you receive any support to help you to control these changes? what help, if any?

Dodosa: Je ulipata msaada wowote wa kukusaidia kudhibiti mabadiliko haya? msaada gani?kama upo

10. Has your health related to diabetes changed since COVID pandemic started?

Je, afya yako kuhusiana na ugonjwa wa kisukari imebadilika tangu kuanza kwa janga la UVIKO-19?

Probe: What has specifically changed?

Dodosa: Nini hasa kimebadilika?

Uliza:

- Changes in blood glucose level
  Mabadiliko katika kiwango cha sukari kwenye damu
- Emerged health problems such as eye problems, wounds that do not heal well, feeling irritated in the legs or feet.
- Matatizo mapya mfano matatizo ya macho, vidonda ambavyo haviponi vizuri, hisia za kuwashwa kwenye miguu au nyayo.
  -Adhere to your medicine (how?)
- Kuzingatia utaritbu wa kutumia dawa zako (vipi?)

-Fear about diabetes illness (if so, why/) what were you afraid of
Hofu kuhusu ugonjwa wa kisukari (ikiwa ni hivyo, kwa nini?) ulikuwa unahofia nini?

- Depression? (If yes,how depression has affected the way you were and how you are coping with your Diabete)
  Msongo wa mawazo? (Ni jinsi gani msongo wa mawazo umeadhiri namna ambavyo ulivyokuwa na unakabiliana na ugonjwa wako wa Kisukari)

Dodosa:

a) Probe: What do you think has contributed to these changes?
   Unadhani nini kisababisha mabadiliko haya?

b) Probe: how have you been handling these changes? has it been easy/difficult?
   Umekuwa ukishughulikiaje mabadiliko haya? imekuwa rahisi/vigumu?

   Probe: have you been worried about these changes? why?
   Dodosa: je umekuwa na wasiwasi kuhusu mabadiliko haya? kwa nini?

Think about the things that may have made you to access to diabetes treatment easier or harder during COVID-19 (after March 2020)

Fikiri kuhusu mambo ambayo huenda yalifanya kupata huduma za matibabu ya kisukari kuwa rahisi au ngumu wakati wa UVIKO-19 (baada ya Machi 2020)

11. Was it difficult/easy was it for you to control diabetes during COVID-19? (After March 2020)

Je ilikuwa ngumu/rahisi vipi kwako kudhibiti ugonjwa wa kisukari katika kipindi cha UVIKO-19? (Baada ya Machi 2020)

Probe: What exactly was difficult for you to control in your diabete? Explain

Dodosa: Ni nini hasa kilikuwa kigumu kwako katika kudhibiti ugonjwa wako wa kisukari? Eleza

Probe: How did you manage to deal with this difficult situation? Was it easy for you to handle this difficulty?

Dodosa: je umewezaje kukabiliana na hali hii ngumu? Je imekuwa rahisi kwako kushughulikia ugumu huu? Je, ulipata msaada katika kudhibiti hali hii ngumu?

Probe: Did you get help in managing this difficult situation?Explain more

Dodosa: je ulipata msaada katika kudhibiti ugonjwa wako wa kisukari? Tuelezee Zaidi

Ask:can you tell me if you experienced any of the following problems?

Uliza: unaweza kuniambia kama ulipata matatizo yanayohusiana na haya yafuatayo?

- Transport, costs, access to health care - how?
  Usafiri, gharama, upatikanaji wa huduma za afya - kivipi?
- Access to medicine - how?
  Upatikanaji wa dawa - vipi?
- Adhere to medica advise, how?
- Kuzingatia ushauri wa matibabu? - vipi?
- Depression? What exactly were you thinking?
  Msongo wa mawazo? Ulikuwa unawaza nini hasa?
- Fear? what exactly were you afraid of?
  Hofu? ulikuwa unahofia nini hasa?

12. **What support have you been getting in managing your diabetes? (*Remind participant that you are referring to the period AFTER MARCH 2020, during COVID-19*)**

Je umekuwa ukipata msaada gani katika kudhibiti ugonjwa wako wa kisukari?*(mkumbushe mshiriki kuwa unarejea kipindi cha* ***BAADA YA MACHI 2020,*** *wakati wa UVIKO-19)*

Probe: Did anyone help you in controlling your diabetes during COVID-19?

Dodosa: Je, kuna mtu yeyote aliyekupa msaada katika kudhibiti kisukari chako wakati wa UVIKO-19?

Ask: family member, health care provider, traditional healer).Tell us more
Uliza:mwanafamilia, mhudumu wa afya, waganga wa kienyeji).Tulezee zaidi

Probe: Did that support help? how that support helped you?
Dodosa**:** Je, msaada huo ulikusaidia? Ulikusaidiaje?

13. In your opinion, what can help diabete patients to manage their illness effectively during the COVID-19 epidemic? [Instructions: You can refer to the diabetes control measure/ways the respondent mentioned earlier]

    Kwa maoni yako ni kitu gani kinaweza kuwasaidia wagonjwa wa kisukari kudhibiti ugonjwa wao ipasavyo wakati wa janga la UVIKO-19? [Maagizo: Unaweza kurejea njia za udhibiti wa kisukari mhojiwa alitaja hapo awali]

We have talked about how to control your diabetes. Now let's talk about **the AVAILABILITY** of diabetic health care that you may have accessed in the pre-COVID-19 period

Tumezungumza juu ya jinsi ya kudhibiti ugonjwa wako wa kisukari. Sasa hebu tuzungumze kuhusu **UPATIKANAJI** wa huduma za afya za kisukari ambazo unaweza kuwa umefikia katika kipindi cha kabla ya UVIKO-19

14. **Was it difficult / easy for you to get diabete health services during COVID-19? (after March 2020)(why?)**
    Je, ilikuwa vigumu/rahisi kwako kupata huduma za afya za ugonjwa wa kisukari katika kipindi cha UVIKO-19? (baada ya Machi 2020) (kwanini?)

    Probe: What exactly was the difficulty in getting diabetes care during COVID-19? How?
    Dodosa: ni jambo gani lilikuwa kigumu katika kupatikanaji wa huduma za ugonjwa wa kisukari wakati wa UVIKO-19? Ki Vipi?

    Probe: How did you manage to deal with this difficult situation? Was it easy for you to handle this difficulty? Have you found help in managing this difficult situation?
    Dodosa: umewezaje kukabiliana na hali hii ngumu? Je ilikuwa rahisi kwako kushughulikia ugumu huu? Je, umepata usaidizi katika udhibiti wa hali hii ngumu?

Probe:Did you get help getting diabetes services during this COVID-19? Tell us more
Dodosa: je umekuwa ukipata msaada katika kupata huduma za matibau ya ugonjwa wa kisukari wakati wa huu wa UVIKO-19? Tuelezee zaidi

Think about the things that may have contributed to access diabetes services easier or difficulty during COVID-19 (after March 2020)

Fikiria kuhusu mambo ambayo huenda yamesababisha kufikia huduma za ugonjwa wa kisukari kuwa rahisi au ngumu katika kipindi cha UVIKO-19 (baada ya Machi 2020)

Ask: Can you tell me exactly if you are experiencing any challenges in accessing diabetes services during COVID-19?
Uliza: Je unaweza kuniambia haswa iwapo unakutana na changamoto zozote katika kupata huduma za ugonjwa wa kisukari wakati wa UVIKO-19?
Probe the following challenges are below:
Dodosa changamoto zifuatazo hapa chini:

- Transport, costs, access to services - how?
  Usafiri, gharama, upatikanaji wa huduma - vipi?
- Access to medicine - how?
- Upatikanaji wa dawa - vipi?
- Adhering to medical advice? - how?
- Kuzingatia ushauri wa matibabu? - vipi?
- Family support
  Msaada wa familia
- Fear / stress, how
  Hofu/ dhiki, vipi?

15. What medical suport did you receive in managing/controlling your diabetes during COVID-19?
Je umepokea msaada gani wa matibabu ya kiafya ulioyopata katika kudhibiti ugonjwa wako wa kisukari wakati wa UVIKO-19?

Probe: Where did you get diabetes care during COVID-19?
Dodosa: Ulipata wapi huduma ya ugonjwa wa kisukari wakati wa UVIKO-19?
Probe: How useful were the services you received in managing diabetes during COVID-19? Tell me more
Dodosa: Je, huduma ulizopokea katika kudhibiti ugonjwa wa kisukari wakati wa UVIKO zilikufaa kiasi gani? Niambie Zaidi

Probe: Did you receive any additional services to help control/manage diabetes during COVID-19 as opposed to starting BEFORE COVID-19? Is this important? Why?

Dodosa: Je ulipata huduma zozote za ziada kukusaidia udhibiti ugonjwa wa kisukari wakati wa UVIKO-19 tofauti na mwanzo KABLA ya UVIKO-19? Je hili ni la muhimu? Kwa nini?

Probe: Has health care been able to help diebetes patients access their services during this COVID-19 period? If so why not? if not why not
Dodosa: Je huduma za afya zimeweza kuwasaidia wenye ugonjwa wa kisukari kupata huduma zao wakati huu wa UVIKO-19? Kama ndiyo ni kwanini? kama sio ni kwa nini?

16. What do you think should be done to help diabete patients to better manage their illness during health events like COVID-19? what exactly should be done?
Je nini unafikiri kifanyike ili kuwasaidia wagonjwa wenye kisukari kudhibiti vizuri ugonjwa wao wakati wa majanga ya kiafya kama UVIKO-19? nini hasa kifanyike?

Section D: Health information and Knowlege about COVID-19 pandemic and Practices

Sehemu D: Taarifa za afya,Maarifa na Uzoefu/Mbinu kuhusu janga la UKIVO-19

Knowledge about COVID-19

<u>Ufakamu kuhusu UVIKO-19</u>

18.Can you tell me about your reaction when you heard about COVID-19?
Je, unaweza kuniambia ulijisikiaje uliposikia kuhusu UVIKO- 19?

19.Where did you hear about UVIKO-19?
Ulisikia wapi kuhusu UVIKO-19?
20.How is COVID-19 transmitted?
Je UVIKO-19 inaambukizwa vipi?
21.How COVID-19 is prevented?
Je UVIKO-19 inaepukwa vipi?
22. What have you personally been doing to avoid getting COVID-19?
Probes
Je wewe mwenyewe umekuwa ukifanya nini ili kuepuka UVIKO-19?
Probes

- **How easy / difficult has it been for you to wear masks?**
  Je imekuwa rahisi/vigumu vipi kwako kuvaa barakoa?
- Has it been easy / hard for you to clean yourself?
  imekuwa rahisi/ngumu kwako kujitakasa?
- How easy / difficult has it been for you to keep social distance?
  Je imekuwa rahisi/vigumu vipi kwako kukaa kwa umbali katika kijamii?
- Did you use any natural remedies to protect yourself from COVID-19?
  Je, ulitumia dawa zozote za asili kujikinga na UVIKO-19?
- If so, ask what were those remedies ?
  Ikiwa ndiyo, uliza dawa hizi zilikuwa ni zipi?

- To what extent the natural remedies helped?
- Je, kwa kiasi gani dawa hizi za asili zinasaidia ?
- were these drugs available?
- Je dawa hizi zilipatikana?
- How did you know these natural remedies?
- Je ulizijuaje dawa hizi?

If not, ask why respondent did not use these traditional remedies?
Ikiwa hapana, uliza kwa nini haukutumia dawa hizi za kienyeji?

23. Do you think COVID-19 can affect your diabetes? if so, explain to me a little bit?
Je UVIKO-19 inaweza kuathiri ugonjwa wako wa kisukari? kama ndiyo nieleze kidogo?

Information about COVID-19
Taarifa kuhusu UVIKO-19

24. Please tell me more about any information you have heard or received about your diabetes management since the COVID-19 epidemic began

Tafadhali niambie zaidi kuhusu taarifa yoyote uliyosikia au kupokea kuhusu udhibiti wa ugonjwa wako wa kisukari tangu janga la UVIKO-19 lianze?

Probes: a

a. Were you specifically informed about how people with diabetes should manage their illness to protect themselves from COVID-19?

Dodosa: Je, ulipewa taarifa namna ambavyo watu wenye ugonjwa wa kisukari wanapaswa kudhibiti ugonjwa wao ili kujikinga na UVIKO-19?

What did the information mean?
Je, taarifa hizo zilihusu nini?
Where did you get the information?
Umepata wapi taarifa?

b. How helpful were the information you received in controlling your diabetes?
Je, taarifa ulizopokea zilikuwa na manufaa kiasi gani kwako katika kudhibiti ugonjwa wako wa kisukari?

c. Did you know about where you can ask questions or get advice for your diabetes control or any other health condition you had during this COVID-19?
Je, ulikuwa unafahamu kuhusu ni wapi unapoweza kuuliza maswali au kupata ushauri kwa ajili ya udhibiti ungonjwa wako wa kisukari au hali yoyote yakiafya uliyokuwa nayo wakati wa huu UVIKO-19?

Impacts of COVID -19 and diabetes
Athari za UVIKO-19 na ugonjwa wa kisukari

In this section we aim to better understand your views on the effects of the COVID-19 pandemic and diabetes

Katika sehemu hii tuna lengo la kuelewa zaidi maoni yako kuhusu athari za janga la UVIKO-19 na ugonjwa wa kisukari

25. Since COVID-19 started, have you had any other health problems?
Tangu UVIKO-19 ilipoanza, je, umepata mtatizo mengine ya kiafya?

Probe:

a. If so, please explain briefly about these health problems
Ikiwa ndiyo, tafadhali elezea kwa ufupi kuhusu matatizo hayo ya kiafya

b. Ask about other problems such as:
Uliza kuhusu matatizo mengine kama vile:
- stress, lack of sleep, inability to interact with people others, fear of COVID or death, mental stress
- mfadhaiko, ukosefu wa usingizi, uwezo wa kushirikiana na watu wengine, hofu itokanayo na UVIKO au kifo, msongo wa mawazo
- concerns about household issues eg access to food, electricity, water, family care
wasiwasi wa maswala ya nyumbani mfano kuhusu upatikanaji wa chakula, umeme, maji, utunzaji wa familia
- income (employment /income-generating activities)any other?
- mapato (kazi/ajira; shughuli za kujiongezea kipato)
- nyingine yoyote?

c. Ask: what respondent think cause that situation?
Muulize: Ni kitu gani anafikiri kinaweza kuwa kilisababisha hali kama hizo

d. Ask how the respondent dealt with that situation and how?
Uliza ikiwa aliweza kukabiliana na hali hizo na jinsi gani?

e. Ask if these conditions have affected the patient's control over diabetes
Uliza kama hali hizi zimeathiri jinsi mgonjwa alivyodhibiti ugonjwa wa kisukari

f. Ask if these conditions have affected the diabetes treatment services provided
Uliza iwapo hali hizi zimeathiri huduma za matibabu ya ugonjwa wa kisukari iliyotolewa?

26. How has diabetes affected your COVID-19 management?
Je, kwa namna gani ugonjwa wa kisukari umeathiri usimamizi/udhibiti wako kwenye UVIKO-19?

Section F: Opinions about COVID-19 Vaccine
Sehemu F: Maoni kuhusu Chanjo ya UVIKO-19
I would also like to know your views on the COVID-19 vaccine
Ningependa pia kujua maoni yako kuhusu chanjo ya UVIKO-19

27. What is your opinion on the COVID-19 vaccine?
- **Je, una maoni gani kuhusu chanjo ya COVID-19?**
  Probe: is vaccine good or bad and why?
  Dodosa: je chanjo ni nzuri au mbaya na kwanini?

28. Have you received the COVID-19 vaccine?
Je, umepata chanjo ya UVIKO-19?
If so, what is it that has motivated you?
Kama ndiyo, ni kitu gani kimekuhamasisha?
If not, what are some of the things that keep you from getting vaccinated**?**
Ikiwa hapana, ni mambo gani yanayozuia usikubali kupata chanjo?

29. Is there anything you would like to ask or talk about about diabetes that we have not discussed?

Je, kuna kitu chochote ambacho ungependa kuuliza au kuzungumzia kuhusu ugonjwa wa kisukari ambacho hatujajadili?

Reseacher: Thanks the participant for his /her valuable time and asks if they have any questions

Mtafiti: Anashukuru mshiriki kwa muda wake wa thamani na kuuliza kama wana swali lolote

...................................END/ MWISHO...
