# Supplementary material for: Factors associated with COVID-19 vaccine uptake among people with type 2 diabetes in Kenya and Tanzania: a mixed-methods study
Source: BMJ Open. 2023 Dec 7;13(12):e073668. doi: 10.1136/bmjopen-2023-073668 (PMC10711896; doi:10.1136/bmjopen-2023-073668)
Supplement: Author's manuscript [file bmjopen-2023-073668.draft_revisions.pdf]

BMJ Open is committed to open peer review. As part of this commitment we make the peer review history of every article we publish publicly available.

When an article is published we post the peer reviewers' comments and the authors' responses online. We also post the versions of the paper that were used during peer review. These are the versions that the peer review comments apply to.

The versions of the paper that follow are the versions that were submitted during the peer review process. They are not the versions of record or the final published versions. They should not be cited or distributed as the published version of this manuscript.

BMJ Open is an open access journal and the full, final, typeset and author-corrected version of record of the manuscript is available on our site with no access controls, subscription charges or pay-per-view fees (http://bmjopen.bmj.com).

If you have any questions on BMJ Open's open peer review process please email info.bmjopen@bmj.com

# Factors associated with COVID-19 vaccine uptake among people with type 2 diabetes in Kenya and Tanzania: a mixed-methods study

| | |
|---|---|
| Journal: | *BMJ Open* |
| Manuscript ID | bmjopen-2023-073668 |
| Article Type: | Original research |
| Date Submitted by the Author: | 14-Mar-2023 |
| Complete List of Authors: | Binyaruka, Peter; Ifakara Health Institute, Department of Health System, Impact Evaluation and Policy<br>Mtenga, Sally M.; Ifakara Health Institute, Health Systems, Impact Evaluation and Policy<br>Mashasi, Irene; Ifakara Health Institute, Health Systems, Impact Evaluation and Policy<br>Karugu, Caroline; African Population and Health Research Center, Chronic Disease Management Unit<br>Mohamed, Shukri; African Population and Health Research Center, Chronic Disease Management Unit<br>Asiki, G; African Population and Health Research Center, Chronic Disease Management Unit<br>Mair, Frances; University of Glasgow, School of Health and Wellbeing<br>Gray, Cindy; University of Glasgow, School of Social and Political Sciences; University of Glasgow, School of Health and Wellbeing |
| Keywords: | COVID-19, Health policy < HEALTH SERVICES ADMINISTRATION & MANAGEMENT, Public health < INFECTIOUS DISEASES, Health Services Accessibility |
| | |

SCHOLARONE™
Manuscripts

*I, the Submitting Author has the right to grant and does grant on behalf of all authors of the Work (as defined in the below author licence), an exclusive licence and/or a non-exclusive licence for contributions from authors who are: i) UK Crown employees; ii) where BMJ has agreed a CC-BY licence shall apply, and/or iii) in accordance with the terms applicable for US Federal Government officers or employees acting as part of their official duties; on a worldwide, perpetual, irrevocable, royalty-free basis to BMJ Publishing Group Ltd ("BMJ") its licensees and where the relevant Journal is co-owned by BMJ to the co-owners of the Journal, to publish the Work in this journal and any other BMJ products and to exploit all rights, as set out in our licence.*

*The Submitting Author accepts and understands that any supply made under these terms is made by BMJ to the Submitting Author unless you are acting as an employee on behalf of your employer or a postgraduate student of an affiliated institution which is paying any applicable article publishing charge ("APC") for Open Access articles. Where the Submitting Author wishes to make the Work available on an Open Access basis (and intends to pay the relevant APC), the terms of reuse of such Open Access shall be governed by a Creative Commons licence – details of these licences and which Creative Commons licence will apply to this Work are set out in our licence referred to above.*

*Other than as permitted in any relevant BMJ Author's Self Archiving Policies, I confirm this Work has not been accepted for publication elsewhere, is not being considered for publication elsewhere and does not duplicate material already published. I confirm all authors consent to publication of this Work and authorise the granting of this licence.*

For peer review only

# Factors associated with COVID-19 vaccine uptake among people with type 2 diabetes in Kenya and Tanzania: a mixed-methods study

Peter Binyaruka[1#*], Sally Mtenga[1#], Irene Mashasi[1], Caroline Karugu[2], Shukri F. Mohamed[2], Gershim Asiki[2], Frances S. Mair[4], & Cindy M. Gray[3,4]

**Author affiliations**

[1] Department of Health system, Impact Evaluation, and Policy, Ifakara Health Institute, PO Box 78373, Dar es Salaam, Tanzania.

[2] Chronic Disease Management Unit, African Population and Health Research Center (APHRC), Nairobi, Kenya.

[3] School of Social and Political Sciences, University of Glasgow, Glasgow, UK

[4] School of Health and Wellbeing, University of Glasgow, Glasgow, UK

*** Corresponding author:** Peter Binyaruka, *pbinyaruka@ihi.or.tz, +255 (0) 655 363361*

[#] **Joint first authors:** Peter Binyaruka, Sally Mtenga

**E-mail addresses:**

Peter Binyaruka: pbinyaruka@ihi.or.tz

Sally Mtenga: smtenga@ihi.or.tz

Irene Mashasi: imashasi@gmail.com

Caroline Karugu: ckarugu@aphrc.org

Shukri F. Mohamed: smohamed@aphrc.org

Gershim Asiki: gasiki@aphrc.org

Frances S. Mair: Frances.Mair@glasgow.ac.uk

Cindy M. Gray: Cindy.Gray@glasgow.ac.uk

**Word Count:** 3152 words

**Key words:** COVID-19, vaccine uptake, determinants, barriers, facilitators, hesitancy, Tanzania.

For peer review only

**Abstract (250 words)**

**Background:** People with type 2 diabetes (T2D) are at increased risk of poor outcomes from COVID-19. Vaccination can improve outcomes, but vaccine hesitancy remains a major challenge. We examined factors influencing COVID-19 vaccine uptake among people with T2D in two sub-Saharan Africa countries that adopted different national approaches to combat COVID-19, Kenya and Tanzania.

**Methods:** A mixed-methods study, involving a survey of 1000 adults with T2D (500 Kenya; 500 Tanzania) and 51 in-depth interviews (21 Kenya; 30 Tanzania). Determinants of COVID-19 vaccine uptake were identified using multivariate logistic regression, while thematic content analysis explored barriers and facilitators.

**Results:** COVID-19 vaccine uptake was lower in Tanzania (26%) than in Kenya (75%), which may reflect an initial political hesitancy about vaccines in Tanzania. People with college/university education were four times more likely to be vaccinated than those with no education (Kenya AOR=4.25 [95%CI:1.00-18.03], Tanzania AOR=4.07 [1.03-16.12]); and people with health insurance were almost twice as likely to be vaccinated than those without health insurance (Kenya AOR=1.70 [1.07-2.70], Tanzania AOR=1.81 [1.04-3.13]). Vaccine uptake was higher in older people in Kenya, and among those with more comorbidities and higher socioeconomic status in Tanzania. Interviewees reported that wanting protection from severe illness promoted vaccine uptake, while conflicting information, misinformation and fear of side-effects limited uptake.

**Conclusion:** COVID-19 vaccine uptake among people with T2D was suboptimal, particularly in Tanzania, where initial political hesitancy had a negative impact. Policymakers must develop strategies to reduce fear and misconceptions, especially among those who are less educated, uninsured, and younger.



For peer review only - http://bmjopen.bmj.com/site/about/guidelines.xhtml

## Key questions

### What is already known?

- People with type 2 diabetes (T2D) are at increased risk of poor outcomes from COVID-19.
- COVID-19 vaccination can reduce incidence of COVID-19, hospitalisation, and improve outcomes, but vaccine hesitancy remains a major challenge.
- We know little about factors influencing COVID-19 vaccine uptake among people with T2D in two sub-Saharan Africa countries.

### What are the new findings?

- Lower vaccine uptake in Tanzania (26%) than in Kenya (75%) reflecting partly the initial political hesitancy about COVID-19 vaccine.
- Being educated and having health insurance associated with higher vaccine uptake in Kenya and Tanzania.
- Vaccine uptake was higher in older people in Kenya, and among those with more comorbidities and higher socioeconomic status in Tanzania.
- Interviewees reported that wanting protection from severe illness promoted vaccine uptake, while conflicting information, misinformation and fear of side-effects limited uptake.

### What do the new findings imply?

- COVID-19 vaccine uptake by people with T2D remains suboptimal, particularly in Tanzania.
- Policymakers and other stakeholders need to provide consistent evidence-based messaging and targeted strategies (e.g., health education) to improve vaccination coverage among different sub-groups of people with T2D and other health conditions, including those who are less educated, uninsured, and younger.



For peer review only - http://bmjopen.bmj.com/site/about/guidelines.xhtml

**INTRODUCTION (3152 words)**

The COVID-19 pandemic has negatively impacted social, health and economic welfare globally. In the health sector, COVID-19 led to healthcare disruptions affecting routine care, especially for patients living with chronic or non-communicable diseases (NCDs) like type 2 diabetes (T2D).[1] People with T2D have a compromised immune system and are at higher risk of infection and adverse outcomes from COVID-19.[2 3]

Vaccination is a proven pharmaceutical intervention to reduce COVID-19 transmission and adverse outcomes.[4] In December 2020, newly developed COVID-19 vaccines were authorised, and efforts by the COVID-19 Vaccines Global Access (COVAX) initiative aimed to ensure global vaccine equity. However, vaccination coverage is still suboptimal in many low-and middle-income countries (LMICs) due to limited COVAX vaccine supply[5] and vaccine hesitancy because of concerns about life-threatening side effects, risk of new diseases, and infertility.[6-10]

High vaccine coverage is required to reduce incidence of COVID-19 and hospitalisation. In many countries, people with chronic diseases, such as T2D, are prioritised during national COVID-19 vaccine rollouts.[11] It is therefore important for policymakers to understand the prevalence of COVID-19 vaccine uptake and factors influencing uptake among people with chronic diseases. A few studies among people with T2D, including only two from sub-Saharan Africa, revealed that vaccine uptake was significantly higher among men, public servants, urban populations and people with a higher education level.[12-20] This paper extends the evidence base by estimating the prevalence of COVID-19 vaccine uptake among people with T2D in Kenya and Tanzania, and identifying the factors influencing uptake in both countries. We chose these two countries because they took different approaches to combat COVID-19 (e.g., 2020 lockdown in Kenya, but not in Tanzania; initial government hesitancy about COVID 19 vaccines in Tanzania, but not in Kenya). We focused on T2D because it is a risk factor for other chronic illness such as kidney failure, cardiovascular disease and hypertension,[21] and increases vulnerability and severity of COVID-19.[2 3] Our findings will guide targeted communication campaigns and other strategies to improve vaccination coverage, especially among people with chronic diseases, such as T2D.



For peer review only - http://bmjopen.bmj.com/site/about/guidelines.xhtml

For peer review only

## METHODS

### Study setting

This study was conducted in in two LMICs in East Africa, Kenya and Tanzania. We purposively selected four counties (two urban – Nairobi, Kiambu; two rural – Vihiga, Nyeri) out of 47 counties in Kenya; and purposively selected two regions (one urban – Dar es Salaam; one rural – Morogoro) out of 31 regions in Tanzania. The prevalence of diabetes mellitus was relatively lower in Kenya (2.4% in 2015)[22] than in Tanzania (9.1% in 2012).[23] COVID-19 vaccination is free in both countries, but priority was initially given to health workers, older and people with chronic health conditions. Both countries received vaccines through COVAX, but the vaccination rollout began slightly earlier in Kenya (March 2021)[24] compared to Tanzania (July 2021).[25]

### Study design

We use a mixed-methods study design involving a cross-sectional survey of 500 people with T2D in each country, and qualitative in-depth interviews with 21 and 30 people with T2D in Kenya and Tanzania, respectively.

### Study participants and sampling

The survey included adults (18+ years) who had received a diagnosis of T2D before COVID-19 (i.e., before March 2020) in Nairobi (n=276), Kiambu (n=104), Vihiga (n=76), and Nyeri (n=44) counties in Kenya, and in Dar es Salaam (n=300) and Morogoro (n=200) regions in Tanzania. Those who were eligible were identified from health facility outpatient registers and approached for written informed consent. Based on convenience sampling, participants for the in-depth interviews were mainly selected from survey participants; however, a few additional people with T2D who were not part of the survey were also interviewed.  People with T2D who were vaccinated or unvaccinated, and with or without other health conditions were eligible to take part in both the survey and in-depth interviews.

### Data collection and recruitment



For peer review only - http://bmjopen.bmj.com/site/about/guidelines.xhtml

Trained fieldworkers administered structured survey questionnaires in both countries and used an interview topic guide to explore facilitators and barriers to COVID-19 vaccination. Both tools were piloted and refined before use. Data collection was conducted in February-March 2022. In Tanzania, all data collection was conducted face-to-face. In Kenya, the survey was administered by phone due to national guidelines to minimise COVID-19 transmission, while the decision was taken to conduct the qualitative interviews face-to-face as they involved a much smaller sample size, and thus lower risk.

**Measures**

Our primary outcome of interest was COVID-19 vaccine uptake among people with T2D in Kenya and Tanzania and was assessed using the following question: *'Have you been vaccinated for COVID-19?'*. We used a binary outcome with a value of 1 if a participant confirmed having received any COVID-19 vaccine (regardless of dosage) by February 2022, and 0 otherwise.

The explanatory variables as potential determinants of COVID-19 vaccine uptake were based on the World Health Organization's (WHO) social determinants of health conceptual framework[26] and relevant empirical evidence on factors influencing health care seeking behaviour, particularly vaccination.[27] We used two categories of determinants: socio-demographic and health-related.

*Socio-demographic factors* included five binary variables: place of residence (rural/urban), sex (male/female), marital status (married/not married), health insurance (insured/non-insured), and socioeconomic status (SES) based on a ladder scale (1-10) (lower 1-5/higher 6-10). We also included four categorical variables: age (<40/40-49/50-59/60-69/>70 years), education level (no education/primary/secondary/higher education), religion (Catholic/Protestant/Muslim), and occupation status (formal sector workers/farmers/self-employed/retired/unemployed).

*Health-related factors* included three binary variables: family history of T2D (history/no history), time living with T2D (<6 years/≥6 years), and presence of comorbidities (with/without comorbidities). The number of comorbidities was assessed by asking participants to report additional long-term conditions.

**Data analysis**



For peer review only - http://bmjopen.bmj.com/site/about/guidelines.xhtml

Descriptive analysis was undertaken to describe participants' socio-demographic and health-related characteristics and prevalence of COVID-19 vaccination in each country. To identify the independent determinants of vaccine uptake, we performed separate multivariate logistic regressions for each country. Since vaccine uptake was higher in Kenya (75% - reflecting a non-rare event), we checked the sensitivity of the logistic regression by applying a modified Poisson regression model.[28] We assessed multicollinearity between independent variables using a pairwise correlation analysis. All correlation coefficients were below 0·5, which is considered moderate, supporting the inclusion of all variables in the regression analysis. STATA version 16 was used to analyse the quantitative data.

Audio-recorded data from the qualitative interviews were transcribed verbatim in Kenya and Tanzania. Researchers repeatedly reviewed the transcripts to familiarize themselves with the data. Thematic content analysis was employed to identify common phrases related to the factors that facilitated and deterred COVID-19 vaccine uptake. In this process, inductive coding was used independently by researchers from each study country to categorise the key themes. They then met to discuss and agree on the codes that were applied to all transcripts. NVivo software (version 10 in Kenya, version 12 Tanzania) was used to manage and analyse the data.

**Patient and public involvement:** Patients and/or the public were not involved in the design, or conduct, or reporting, or dissemination plans of this research.

## RESULTS

### Participant characteristics

Most participants in both countries were from urban settings (>59%), female (>65%), and married (>62%) (Table 1). The majority were also aged above 50 years (the average age was 58 years in Kenya and 57 years in Tanzania) and had completed primary or secondary education. However, religious affiliation differed between the two countries, with almost equal numbers of Muslims and Christians in Tanzania, while Christians dominated in Kenya. In both countries, the majority of participants worked as farmers or were self-employed, had health insurance (>60%), and were of lower SES (89% in Kenya, 75% in Tanzania). Fewer participants in Tanzania (42%) had a family history of T2D than in Kenya (53%). Most patients

For peer review only

in both countries had lived with T2D for 6 years or more (>61%), while the mean number of comorbidities was 3.4 in Kenya and 1.5 in Tanzania.

Most people who participated in the qualitative in-depth interviews had comorbidities (particularly hypertension). They were aged between 60 and 80 years in Kenya, and between 46 and 51 years in Tanzania. In urban areas, interviewees were employed/running businesses, retired or unemployed, while in rural areas the majority were farmers.

**Factors influencing COVID-19 vaccine uptake**

The survey data showed that COVID-19 vaccine uptake was relatively lower in Tanzania than in Kenya. Only 26% of participants in Tanzania reported having received at least one COVID-19 vaccination, compared with 75% in Kenya. The qualitative interviews suggested that the low rate of vaccine uptake in Tanzania may reflect the political approach to COVID-19 in that country. Initially, the past presidential regime promoted natural remedies like lemon, ginger, and steam with herbal remedies to combat COVID-19, as well as WHO recommended prevention measures (e.g., masks and hands sanitizer), but did not endorse COVID-19 vaccination. Despite the current Tanzanian government's support and promotion of COVID-19 vaccination rollout from July 2021, the initial political hesitancy emerged as a continued barrier to vaccine uptake in the accounts of Tanzanian interviewees.

> *"I cannot say the COVID-19 vaccine is good because our late President said the vaccine cannot protect us from COVID-19 rather we should protect ourselves and said absolutely there is no vaccine to cure COVID-19, so we are following him"* **[Female-PT09-urban-Tanzania]**.

The survey data showed that participants with a college/university education were over four times more likely to be vaccinated than those with no education in Kenya (AOR=4.25, 95%CI: 1.00-18.03, $p<0.05$) and Tanzania (AOR=4.07, 95%CI: 1.03-16.12, $p<0.05$) (Table 2). The qualitative interviews revealed the importance of education, particularly in terms of understanding and weighing up the complex information available about COVID-19. For instance, some patients who were vaccinated in both countries felt that protecting themselves from the risk of severe illness outweighed any perceived risk from the COVID-19 vaccine.

> *"I just think it is good. I took AstraZeneca and it did not affect me in any way. In fact, it gave me a sense of safety. You feel you are a little bit safe"* **[Female-220312_1439-rural-Kenya]**



For peer review only - http://bmjopen.bmj.com/site/about/guidelines.xhtml

However, some interviewees also described the difficulty people faced in trying to navigate the diverse and sometimes conflicting information from different sources, such as government leaders, religious leaders, and health care professionals. Some participants reported using wider evidence, such as the international response to COVID-19, to inform their decision to get vaccinated:

> *"I feel like what the government is saying, other countries are also doing it, I don't see how it can be a bad thing"* **[Female-220312_1439-rural-Kenya].**

In addition to formal sources of information, people in both countries also used informal local knowledge, which was not always helpful, to inform their decision-making. Interviewees, particularly in rural Tanzania, described how myths and misconceptions about the COVID-19 vaccine in their communities increased vaccine hesitancy locally:

> *"People say if you vaccinate you can become vampire-like and later you will be like a zombie! You can even give birth to an abnormal child"* **[Female-PT12-rural Tanzania].**

These myths and misconceptions were fuelled by the speed of the development and rollout of COVID-19 vaccines. Interviewees in both countries, expressed concerns about vaccine quality and safety, including the potential of adverse side effects. For, example one man from rural Kenya described how the fear was intensified for many people with T2D:

> *"I think even now most diabetic people are afraid of getting the vaccine. I was also afraid at first because it was believed that if you get vaccinated and you have diabetes you will die"* **[Male-220312_1220-rural-Kenya].**

Health insurance emerged as another strong predictor of vaccine uptake in the quantitative analysis: people with health insurance were almost twice as likely to be vaccinated than those who were not insured in Kenya (AOR=1.70, 95%CI: 1.07-2.70, $p<0.05$) and Tanzania (AOR=1.81, 95%CI: 1.04-3.13, $p<0.05$) (Table 2). The interviews suggested that the lower vaccine uptake among participants without health insurance may in part reflect the fact that the financial hardship caused by the pandemic meant that accessing healthcare for those who were not insured was less important than other competing priorities:



For peer review only - http://bmjopen.bmj.com/site/about/guidelines.xhtml

*"I run a small business, so, whatever I get I put it aside to feed my children, and some to buy my diabetic treatment because I don't have health insurance. If I had the insurance, it would have been easier, I could just come to the hospital and I will be given medication. But now I need to buy (medicine) and the economic situation isn't good with this CORONA at hand. If I had the money, I would buy medicines to take me for two weeks or a month, and I would not miss any dose, but now I can't"* [**Female PT03-urban-Tanzania**].

In Kenya, older patients (50-59 years and 60-69 years) had a higher odds of vaccine uptake (AOR=2.51, 95%CI: 1.10-5.72, $p<0.05$) and (AOR=2.48, 95%CI: 1.05-5.84, $p<0.05$), respectively, compared to young patients (<40 years), a similar, but weaker, association was evident Tanzania (Table 2). In Tanzania (but not Kenya), having more comorbidities was associated with higher vaccine uptake (AOR=1.28, 1.01-1.61, $p<0.05$). Being married showed a weak positive association with vaccine uptake in both countries ($p<0.10$), and lower socioeconomic status was weakly associated with lower vaccine uptake in Tanzania only ($p=0.059$). The Poisson regression results (Appendix Table 1) were broadly similar to the logistic regression results. However, the associations between higher education and being married and vaccine uptake disappeared in Kenya.

## DISCUSSION

Our investigation of COVID-19 vaccine uptake among people with T2D found that 75% and 26% reported having had at least one vaccination in Kenya and Tanzania, respectively. Vaccine uptake was lower in Tanzania partly because of the vaccine hesitancy of the past presidential regime by arguing to conduct a robust evaluation before accepting the use of vaccines,[29 30] which continued to influence people's decision making around whether to be vaccinated, despite the subsequent government's promotion of vaccination during the national rollout from July 2021.[25] Being better educated and having health insurance were significantly associated with higher COVID-19 vaccine uptake in both countries. Vaccine uptake was also higher among older people with T2D in Kenya, and among those with increased number of comorbidities in Tanzania. Interviewees reported that concerns about becoming severely ill if they contracted COVID-19 informed their decision to get vaccinated, while fear of vaccine side effects/safety/quality, different and sometimes conflicting information on vaccines, and local myths and misinformation limited vaccine uptake.



For peer review only - http://bmjopen.bmj.com/site/about/guidelines.xhtml

The finding that higher education levels were associated with increased vaccine uptake in both countries is consistent with previous research on COVID-19 vaccination among people with chronic illness including diabetes in Australia[31] and Italy,[13] and with a study showing an association between better education and intention to get the COVID-19 vaccine among chronically ill people in Saudi Arabia.[15] However, a similar study in Sudan showed no association between education and vaccine uptake.[9]

The finding that having health insurance was associated with increased COVID-19 vaccine uptake among people with T2D in both countries is consistent with the results of a study of people with chronic conditions who had health insurance in Ethiopia.[16] Evidence suggests that having health insurance improves people's access to health care services,[32] which may help people with health insurance feel more empowered to access vaccines than those without health insurance.

The fact that in Tanzania, people with more comorbidities were more likely to have been vaccinated likely reflects their perception of increased vulnerability from contracting COVID-19. This interpretation of the quantitative findings is supported by the qualitative accounts that COVID-19 vaccination was thought to offer protection from severe illness. The findings may also reflect the success of the Tanzanian government's strategy to sensitise and prioritise vulnerable population groups during the July 2021 COVID-19 vaccine rollout. However, the number of comorbidities was not associated with COVID-19 vaccine uptake in Kenya, which may reflect the higher overall vaccination coverage.

In our study, being older was associated with higher COVID-19 vaccine uptake in Kenya. This finding is consistent with a study conducted across five sub-Saharan African countries (Burkina Faso, Ethiopia, Ghana, Nigeria, and Tanzania), which reported that vaccine hesitancy was extremely high among young people (in this case adolescents).[33] However, other studies have shown mixed associations between vaccine uptake and age among people with T2D. For instance, age was positively associated with COVID-19 vaccination in Australia,[31] but negatively associated in Saudi Arabia.[34] Higher SES was also weakly associated with increased vaccine uptake in Tanzania, which supports the results of previous studies that included people with T2D in Australia,[31] China,[8] and Ethiopia.[16]

The fear of vaccine side effects, safety, and quality emerged among factors limiting the uptake of COVID-19 vaccination in both countries. These findings are consistent with other literature


For peer review only - http://bmjopen.bmj.com/site/about/guidelines.xhtml

about COVID-19 vaccine hesitancy in LMICs,[6-10] which have highlighted particular concerns about death, developing new diseases, and infertility. Such concerns have been heightened by the rapid development and production of COVID-19 vaccines and the novel mRNA-based vaccine technology.[35]

To our knowledge this is the first large population-based, mixed-methods study to examine COVID-19 vaccine uptake, as well as the factors influencing uptake, among people with T2D in two LMICs with different national approaches to combatting COVID-19. The study offers insights into the impact on vaccine uptake of these different approaches, which included initial political hesitancy towards vaccination in Tanzania which delayed the vaccination rollout, compared to a pro-vaccine view within the Kenyan government. It is also the first study to examine the influence of comorbidities on COVID-19 vaccine uptake in people with T2D.

Our study had some limitations. First, we were unable to get a fully representative sample of people with T2D in each country, because we only recruited those who were diagnosed and had attended care in health facilities. Vaccine uptake may be higher in this sub-group because they are more engaged with health services than those who do not attend local health facilities. Future studies should recruit people with T2D in community settings to include those who are undiagnosed and/or not registered at health facilities. Second, our study assessed vaccine uptake when national rollout had not yet been fully implemented, particularly in Tanzania. However, in both Kenya and Tanzania, people with chronic diseases such as T2D were prioritized, so it is likely that our study participants would all have been offered at least one vaccination. Third, as COVID-19 vaccination status was self-reported, social desirability bias may have inflated reports of being vaccinated.

Our study has important implications for improving COVID-19 vaccine uptake among people with chronic diseases like T2D in countries like Kenya and Tanzania. It highlights the need for governments to engage with and promote evidence-based health advice (such as the effectiveness of COVID-19 vaccines in reducing disease transmission and severity) to encourage vaccine uptake. It also provides a basis to help policymakers in Kenya and Tanzania develop clear policies and strategies to reduce vaccine hesitancy among people with T2D and other chronic diseases. Potential strategies for consideration include national COVID-19 vaccination education campaigns targeting different 'vaccine hesitant' sub-groups, such as those who are less well educated (including low SES), those who do not have health insurance and younger people. However, it is also important to counter the anti-vaccination views,



For peer review only - http://bmjopen.bmj.com/site/about/guidelines.xhtml

perceptions and beliefs that can rapidly undermine efforts to promote vaccine uptake,[36] for example, by using influential leaders and role models in local and national multi-media communication campaigns.

## Ethics

Ethical approval was obtained from respective ethics committees in Kenya and Tanzania. In Kenya, the permit was obtained from the African Population and Health Research Center Ethics Review Committee (ref. no: DOR 2021/041), African Medical and Research Foundation (AMREF) Health Africa's Ethics and Scientific Research Committee (ref. no: AMREF-ESRC P900/2020), and National Commission for Science, Technology and Innovation (licence no: NACOSTI/p22/14986). In Tanzania, the institutional ethical approval was from the Ifakara Health Institute (IHI/IRB/No: 38-2021), while the national approval was from the National Institute of Medical Research (NIMR/HQ/R.8a/Vol.X/3806). We sought written informed consent from all respondents by providing information on the objectives of the research and the procedures applied in a clear language using an information sheet.

**Conflict of interest:** None.

**Authors' contributions:** SM, GA, FM and CMG designed the study. PB and CK analyzed the survey data with support from GA. IM, SM and SFM analyzed qualitative data from both countries. SM, CMG, FM and GA contributed to data interpretation. PB and SM wrote jointly the first draft of the manuscript. CMG, FM and GA supported development of subsequent drafts. All authors approved the final manuscript.

**Funding:** This work was supported by the Medical Research Council and the National Institute for Health Research (NIHR) [MR/V035924/1] using aid from the UK Government to support global health research. The views expressed are those of the author(s) and not necessarily those of the MRC, NIHR, or the UK government.

**Patient consent:** Obtained.

For peer review only

## REFERENCES


1. Chang AY, Cullen MR, Harrington RA, et al. The impact of novel coronavirus COVID-19 on noncommunicable disease patients and health systems: a review. *Journal of internal medicine* 2021;289(4):450-62.
2. Fang L, Karakiulakis G, Roth M. Are patients with hypertension and diabetes mellitus at increased risk for COVID-19 infection? *Lancet Respir Med* 2020;8(4):e21. doi: 10.1016/s2213-2600(20)30116-8 [published Online First: 2020/03/15]
3. Huang I, Lim MA, Pranata R. Diabetes mellitus is associated with increased mortality and severity of disease in COVID-19 pneumonia - A systematic review, meta-analysis, and meta-regression. *Diabetes Metab Syndr* 2020;14(4):395-403. doi: 10.1016/j.dsx.2020.04.018 [published Online First: 2020/04/26]
4. Watson OJ, Barnsley G, Toor J, et al. Global impact of the first year of COVID-19 vaccination: a mathematical modelling study. *The Lancet Infectious Diseases* 2022
5. Wouters OJ, Shadlen KC, Salcher-Konrad M, et al. Challenges in ensuring global access to COVID-19 vaccines: production, affordability, allocation, and deployment. *The Lancet* 2021;397(10278):1023-34.
6. Piltch-Loeb R, Harriman NW, Healey J, et al. COVID-19 Vaccine Concerns about Safety, Effectiveness, and Policies in the United States, Canada, Sweden, and Italy among Unvaccinated Individuals. *Vaccines (Basel)* 2021;9(10):1138.
7. Diaz P, Zizzo J, Balaji NC, et al. Fear about adverse effect on fertility is a major cause of COVID-19 vaccine hesitancy in the United States. *Andrologia* 2022;54(4):e14361.
8. Duan L, Wang Y, Dong H, et al. The COVID-19 Vaccination Behavior and Correlates in Diabetic Patients: A Health Belief Model Theory-Based Cross-Sectional Study in China, 2021. *Vaccines (Basel)* 2022;10(5) doi: 10.3390/vaccines10050659 [published Online First: 2022/05/29]
9. Omar SM, Khalil R, Adam I, et al. The Concern of COVID-19 Vaccine Safety Is behind Its Low Uptake among Patients with Diabetes Mellitus in Sudan. *Vaccines (Basel)* 2022;10(4) doi: 10.3390/vaccines10040527 [published Online First: 2022/04/24]
10. Kabakama S, Konje ET, Dinga JN, et al. Commentary on COVID-19 Vaccine Hesitancy in sub-Saharan Africa. *Tropical Medicine and Infectious Disease* 2022;7(7):130.
11. Liu Y, Sandmann FG, Barnard RC, et al. Optimising health and economic impacts of COVID-19 vaccine prioritisation strategies in the WHO European Region: a mathematical modelling study. *The Lancet Regional Health-Europe* 2022;12:100267.
12. Aldossari KK, Alharbi MB, Alkahtani SM, et al. COVID-19 vaccine hesitancy among patients with diabetes in Saudi Arabia. *Diabetes Metab Syndr* 2021;15(5):102271. doi: 10.1016/j.dsx.2021.102271 [published Online First: 2021/09/10]
13. Guaraldi F, Montalti M, Di Valerio Z, et al. Rate and Predictors of Hesitancy toward SARS-CoV-2 Vaccine among Type 2 Diabetic Patients: Results from an Italian Survey. *Vaccines (Basel)* 2021;9(5) doi: 10.3390/vaccines9050460 [published Online First: 2021/06/03]
14. Asadi-Pooya AA, Barzegar Z, Sadeghian S, et al. COVID-19 Vaccine Hesitancy Among Patients With Epilepsy or Other Chronic Conditions. *Disaster Med Public Health Prep* 2021:1-3. doi: 10.1017/dmp.2021.311 [published Online First: 2021/10/12]
15. Al-Hanawi MK, Ahmad K, Haque R, et al. Willingness to receive COVID-19 vaccination among adults with chronic diseases in the Kingdom of Saudi Arabia. *J Infect Public*




For peer review only - http://bmjopen.bmj.com/site/about/guidelines.xhtml

*Health* 2021;14(10):1489-96. doi: 10.1016/j.jiph.2021.08.002 [published Online First: 2021/08/22]
16. Kassa Mekonnen C, Gizaw Demissie N, Wako Beko Z, et al. Intent to get vaccinated against COVID-19 pandemic and its associated factors among adults with a chronic medical condition. *Int J Afr Nurs Sci* 2022;16:100401. doi: 10.1016/j.ijans.2022.100401 [published Online First: 2022/03/01]
17. Kolobov T, Djuraev S, Promislow S, et al. Determinants of COVID-19 vaccine acceptance among adults with diabetes and in the general population in Israel: A cross-sectional study. *Diabetes Res Clin Pract* 2022;189:109959. doi: 10.1016/j.diabres.2022.109959 [published Online First: 2022/06/18]
18. Osuagwu UL, Langsi R, Ovenseri-Ogbomo G, et al. Analysis of Perception, Reasons, and Motivations for COVID-19 Vaccination in People with Diabetes across Sub-Saharan Africa: A Mixed-Method Approach. *Int J Environ Res Public Health* 2022;19(13) doi: 10.3390/ijerph19137875 [published Online First: 2022/07/10]
19. Scoccimarro D, Panichi L, Ragghianti B, et al. Sars-CoV2 vaccine hesitancy in Italy: A survey on subjects with diabetes. *Nutr Metab Cardiovasc Dis* 2021;31(11):3243-46. doi: 10.1016/j.numecd.2021.09.002 [published Online First: 2021/10/12]
20. Wang Y, Duan L, Li M, et al. COVID-19 Vaccine Hesitancy and Associated Factors among Diabetes Patients: A Cross-Sectional Survey in Changzhi, Shanxi, China. *Vaccines (Basel)* 2022;10(1) doi: 10.3390/vaccines10010129 [published Online First: 2022/01/23]
21. WHO. Diabetes Fact Sheet. 2022; (14th October 2022). https://www.who.int/news-room/fact-sheets/detail/diabetes (accessed 14th October 2022).
22. Mohamed SF, Mwangi M, Mutua MK, et al. Prevalence and factors associated with pre-diabetes and diabetes mellitus in Kenya: results from a national survey. *BMC Public Health* 2018;18(3):1215. doi: 10.1186/s12889-018-6053-x
23. Mayige M, Kagaruki G, Ramaiya K, et al. Non communicable diseases in Tanzania: a call for urgent action. *Tanzan J Health Res* 2011;13(5 Suppl 1):378-86. doi: 10.4314/thrb.v13i5.7 [published Online First: 2011/12/01]
24. Orangi S, Pinchoff J, Mwanga D, et al. Assessing the level and determinants of COVID-19 vaccine confidence in Kenya. *Vaccines (Basel)* 2021;9(8):936.
25. Mwengee W, Kileo N. The United Republic of Tanzania receives the first COVAX shipment WHO Regional Office for Africa: WHO Regional Office for Africa. Available online: https://www.afro.who.int/news/united-republic-tanzania-receives-first-covax-shipment (Accessed on 9th August 2022); 2021 [
26. Solar O, Irwin A. A conceptual framework for action on the social determinants of health. Social Determinants of Health. Discussion Paper 2 (Policy and Practice), 2010.
27. Phillips DE, Dieleman JL, Lim SS, et al. Determinants of effective vaccine coverage in low and middle-income countries: a systematic review and interpretive synthesis. *BMC Health Services Research* 2017;17(1):681. doi: 10.1186/s12913-017-2626-0
28. Zou G. A modified poisson regression approach to prospective studies with binary data. *Am J Epidemiol* 2004;159(7):702-6. doi: 10.1093/aje/kwh090 [published Online First: 2004/03/23]
29. Makoni M. Tanzania refuses COVID-19 vaccines. *The Lancet* 2021;397(10274):566.
30. Mfinanga SG, Mnyambwa NP, Minja DT, et al. Tanzania's position on the COVID-19 pandemic. *The Lancet* 2021;397(10284):1542-43.

For peer review only


For peer review only - http://bmjopen.bmj.com/site/about/guidelines.xhtml

31. Day D, Grech L, Nguyen M, et al. Serious Underlying Medical Conditions and COVID-19 Vaccine Hesitancy: A Large Cross-Sectional Analysis from Australia. *Vaccines (Basel)* 2022;10(6) doi: 10.3390/vaccines10060851 [published Online First: 2022/06/25]
32. Amani PJ, Hurtig AK, Frumence G, et al. Health insurance and health system (un) responsiveness: a qualitative study with elderly in rural Tanzania. *BMC Health Serv Res* 2021;21(1):1140. doi: 10.1186/s12913-021-07144-2 [published Online First: 2021/10/24]
33. Wang D, Chukwu A, Mwanyika-Sando M, et al. COVID-19 vaccine hesitancy and its determinants among sub-Saharan African adolescents. *PLOS Global Public Health* 2022;2(10):e0000611. doi: 10.1371/journal.pgph.0000611
34. Tourkmani AM, Bin Rsheed AM, AlEissa MS, et al. Prevalence of COVID-19 Infection among Patients with Diabetes and Their Vaccination Coverage Status in Saudi Arabia: A Cross-Sectional Analysis from a Hospital-Based Diabetes Registry. *Vaccines (Basel)* 2022;10(2) doi: 10.3390/vaccines10020310 [published Online First: 2022/02/27]
35. van Riel D, de Wit E. Next-generation vaccine platforms for COVID-19. *Nature materials* 2020;19(8):810-12.
36. Prieto Curiel R, González Ramírez H. Vaccination strategies against COVID-19 and the diffusion of anti-vaccination views. *Scientific Reports* 2021;11(1):1-13.

For peer review only


For peer review only - http://bmjopen.bmj.com/site/about/guidelines.xhtml

List of Tables

**Table 1: Socio-demographic and health-related characteristics of T2D patients**

| Variable | Description | Kenya (N=500) | | Tanzania (N=500) | |
|---|---|---|---|---|---|
| | | n | % | n | % |
| Place of residence | Urban | 380 | 76.0% | 300 | 60.0% |
| | Rural | 120 | 24.0% | 200 | 40.0% |
| Sex | Female | 330 | 66.0% | 336 | 67.2% |
| | Male | 170 | 34.0% | 164 | 32.8% |
| Marital status | Married | 319 | 63.8% | 312 | 62.4% |
| | Not married | 181 | 36.2% | 188 | 37.6% |
| Age group | <40 years | 39 | 7.8% | 36 | 7.2% |
| | 40-49 years | 80 | 16.0% | 62 | 12.4% |
| | 50-59 years | 135 | 27.0% | 165 | 33.0% |
| | 60-69 years | 155 | 31.0% | 206 | 41.2% |
| | ≥70 years | 91 | 18.2% | 31 | 6.2% |
| Mean age in years [SD] | | 500 | 58.2 [12.6] | 500 | 56.8 [10.2] |
| Education level | No education | 16 | 3.2% | 34 | 6.8% |
| | Primary education | 208 | 41.6% | 298 | 59.6% |
| | Secondary education | 201 | 40.2% | 126 | 25.2% |
| | Higher education | 75 | 15.0% | 42 | 8.4% |



For peer review only - http://bmjopen.bmj.com/site/about/guidelines.xhtml

| | | | | | |
|---|---|---|---|---|---|
| **Religion** | Catholic | 104 | 20.8% | 137 | 27.4% |
| | Protestants | 379 | 75.8% | 110 | 22.0% |
| | Muslims | 17 | 3.4% | 253 | 50.6% |
| **Occupation** | Formal workers | 31 | 6.2% | 40 | 8.0% |
| | Farmers (small/large scale) | 78 | 15.6% | 103 | 20.6% |
| | Self-employed (small/large business) | 162 | 32.4% | 190 | 38.0% |
| | Retired | 55 | 11.0% | 72 | 14.4% |
| | Unemployed | 174 | 34.8% | 95 | 19.0% |
| **Health insurance** | Insured | 337 | 67.4% | 302 | 60.4% |
| | Not insured | 163 | 32.6% | 198 | 39.6% |
| **Socioeconomic status^** | Lower SES (1–5) | 447 | 89.4% | 374 | 74.8% |
| | Higher SES (6–10) | 53 | 10.6% | 126 | 25.2% |
| **Family history of T2D** | History of T2D | 267 | 53.4% | 205 | 41.0% |
| | No history of T2D | 233 | 46.6% | 295 | 59.0% |
| **Time living with T2D** | <6 years | 191 | 38.2% | 166 | 33.2% |
| | ≥6 years | 309 | 61.8% | 334 | 66.8% |
| **Mean number of comorbidities [SD]** | | 500 | 3.4 [3.9] | 368 | 1.5 [0.8] |

Notes: Urban locations were Nairobi and Kiambu (Kenya) and Dar es Salaam (Tanzania); rural locations were Nyeri and Vihiga (Kenya) and Morogoro (Tanzania); ^ as measured by a self-report ladder scale where 1 represents low SES and 10 high SES.



For peer review only - http://bmjopen.bmj.com/site/about/guidelines.xhtml

**Table 2: Multivariate logistic regression results of potential predictors of COVID-19 vaccine uptake**

| Variable | Description | Kenya | | Tanzania | |
|---|---|---|---|---|---|
| | | AOR (p-value) | [95%CI] | AOR (p-value) | [95%CI] |
| **Place of residence** | Urban (ref) | 1.00 | | 1.00 | |
| | Rural | 1.09 (0.789) | [0.59-2.00] | 0.86 (0.605) | [0.49-1.50] |
| **Sex** | Female (ref) | 1.00 | | 1.00 | |
| | Male | 1.11 (0.696) | [0.65-1.92] | 1.30 (0.309) | [0.78-2.17] |
| **Marital status** | Not married (ref) | 1.00 | | 1.00 | |
| | Married | 1.56 (0.008) | [0.95-2.57] | 1.55 (0.086) | [0.94-2.57] |
| **Age group** | <40 years (ref) | 1.00 | | 1.00 | |
| | 40-49 years | 1.44 (0.402) | [0.61-3.37] | 1.21 (0.761) | [0.35-4.19] |
| | 50-59 years | 2.51 (0.029) | [1.10-5.72] | 1.56 (0.438) | [0.51-4.76] |
| | 60-69 years | 2.48 (0.038) | [1.05-5.84] | 2.11 (0.200) | [0.67-6.61] |
| | > 70 years | 2.46 (0.078) | [0.90-6.71] | 2.19 (0.270) | [0.54-8.82] |
| **Education level** | No education (ref) | 1.00 | | 1.00 | |
| | Primary education | 1.38 (0.596) | [0.42-4.57] | 1.47 (0.510) | [0.47-4.56] |
| | Secondary education | 1.54 (0.490) | [0.45-5.21] | 1.79 (0.345) | [0.53-6.03] |
| | Higher (college/university) education | 4.25 (0.049) | [1.00-18.03] | 4.07 (0.046) | [1.03-16.12] |
| **Religion** | Catholic (ref) | 1.00 | | 1.00 | |
| | Protestants | 0.87 (0.620) | [0.49-1.53] | 1.19 (0.565) | [0.66-2.13] |

For peer review only

| | | | | | |
|---|---|---|---|---|---|
| | Muslims | 0.67 (0.512) | [0.20-2.23) | 0.94 (0.822) | [0.55-1.62] |
| **Occupation status** | Formal workers (ref) | 1.00 | | 1.00 | |
| | Farmers | 2.86 (0.115) | [0.77-10.62] | 1.15 (0.777) | [0.44-3.00] |
| | Self-employed business | 1.33 (0.623) | [0.43-4.14] | 1.16 (0.742) | [0.47-2.87] |
| | Taking care of home | 1.05 (0.952) | [0.20-5.58] | | |
| | Retired | 1.26 (0.735) | [0.33-4.91] | 0.89 (0.809) | [0.34-2.32] |
| | Unemployed | 0.81 (0.728) | [0.26-2.58] | 0.99 (0.991) | [0.35-2.80] |
| **Health insurance** | Insured | 1.70 (0.026) | [1.07-2.70] | 1.81 (0.035) | [1.04-3.13] |
| | Not insured (ref) | 1.00 | | 1.00 | |
| **Socioeconomic status** | Lower SES (1–5) | 0.79 (0.522) | [0.38-1.63] | 0.62 (0.059) | [0.37-1.02] |
| **(ladder scale 1-10)** | Higher SES (6–10) (ref) | 1.00 | | 1.00 | |
| **Family history of T2D** | Yes | 1.42 (0.123) | [0.91-2.21] | 1.35 (0.189) | [0.86-2.10] |
| | No (reference) | 1.00 | | 1.00 | |
| **Time living with T2D** | <6 years | 0.75 (0.228) | [0.47-1.20] | 0.75 (0.249) | [0.46-1.22] |
| | ≥6 years (ref) | 1.00 | | 1.00 | |
| **Comorbidities** | Number of comorbidities | 1.08 (0.691) | [0.75-1.55] | 1.28 (0.042) | [1.01-1.61] |

Notes: AOR=Adjusted Odds Ratio; CI=Confidence Interval.

For peer review only

# Appendix

**Appendix Table 1: Multivariate Modified Poisson regression results of potential predictors of COVID-19 vaccine uptake**

| Variable | Description | Kenya | | Tanzania | |
|---|---|---|---|---|---|
| | | RR (p-value) | (95%CI) | RR (p-value) | (95%CI) |
| **Place of residence** | Urban (ref) | 1.00 | | 1.00 | |
| | Rural | 1.01 (0.900) | (0.90-1.12) | 0.90 (0.554) | (0.63-1.29) |
| **Sex** | Female (ref) | 1.00 | | 1.00 | |
| | Male | 1.02 (0.751) | (0.91-1.14) | 1.17 (0.355) | (0.84-1.63) |
| **Marital status** | Not married (ref) | 1.00 | | 1.00 | |
| | Married | 1.10 (0.118) | (0.98-1.25) | 1.37 (0.085) | (0.96-1.97) |
| **Age group** | <40 years (ref) | 1.00 | | 1.00 | |
| | 40-49 years | 1.15 (0.411) | (0.82-1.62) | 1.16 (0.760) | (0.45-3.00) |
| | 50-59 years | **1.33 (0.071)** | **(0.98-1.82)** | 1.38 (0.463) | (0.59-3.24) |
| | 60-69 years | **1.33 (0.071)** | **(0.98-1.83)** | 1.72 (0.214) | (0.73-4.03) |
| | > 70 years | **1.33 (0.085)** | **(0.96-1.85)** | 1.74 (0.249) | (0.68-4.44) |
| **Education level** | No education (ref) | 1.00 | | 1.00 | |
| | Primary education | 1.07 (0.690) | (0.78-1.46) | 1.41 (0.481) | (0.55-3.62) |
| | Secondary education | 1.09 (0.583) | (0 80-1.50) | 1.63 (0.327) | (0.61-4.36) |
| | Higher education | 1.27 (0.144) | (0.92-1.77) | **2.63 (0.065)** | **(0.94-7.35)** |

For peer review only

| | | | | | |
|---|---|---|---|---|---|
| **Religion** | Catholic (ref) | 1.00 | | 1.00 | |
| | Protestants | 0.97 (0.611) | (0.87-1.09) | 1.11 (0.581) | (0.76-1.61) |
| | Muslims | 0.87 (0.448) | (0.61-1.25) | 0.95 (0.770) | (0.67-1.35) |
| **Occupation status** | Formal workers (ref) | 1.00 | | 1.00 | |
| | Farmers | 1.18 (0.137) | (0.95-1.46) | 1.08 (0.809) | (0.57-2.07) |
| | Self-employed business | 1.05 (0.631) | (0.87-1.26) | 1.10 (0.765) | (0.58-2.08) |
| | Taking care of home | 0.98 (0.900) | (0.69-1.39) | | |
| | Retired | 1.03 (0.825) | (0.82-1.28) | 0.93 (0.819) | (0.49-1.75) |
| | Unemployed | 0.91 (0.384) | (0.73-1.13) | 0.98 (0.949) | (0.46-2.07) |
| **Health insurance** | Insured | 1.14 (0.035) | (1.01-1.29) | 1.56 (0.045) | (1.01-2.41) |
| | Not insured (ref) | 1.00 | | 1.00 | |
| **Socioeconomic status (ladder scale 1-10)** | Lower SES (1–5) | 0.96 (0.637) | (0.82-1.13) | 0.74 (0.075) | (0.53-1.03) |
| | Higher SES (6–10) (ref) | 1.00 | | 1.00 | |
| **Family history of T2D** | Yes | 1.08 (0.130) | (0.98-1.19) | 1.22 (0.189) | (0.91-1.65) |
| | No (reference) | 1.00 | | 1.00 | |
| **Time living with T2D** | <6 years | 0.93 (0.208) | (0.84-1.04) | 0.82 (0.266) | (0.57-1.17) |
| | ≥6 years (ref) | 1.00 | | 1.00 | |
| **Comorbidities** | Number of comorbidities | 1.02 (0.629) | (0.95-1.10) | 1.18 (0.022) | (1.02-1.35) |

For peer review only

BMJ Open

# Factors associated with COVID-19 vaccine uptake among people with type 2 diabetes in Kenya and Tanzania: a mixed-methods study

| | |
|---|---|
| Journal: | *BMJ Open* |
| Manuscript ID | bmjopen-2023-073668.R1 |
| Article Type: | Original research |
| Date Submitted by the Author: | 15-Aug-2023 |
| Complete List of Authors: | Binyaruka, Peter; Ifakara Health Institute, Department of Health System, Impact Evaluation and Policy<br>Mtenga, Sally M.; Ifakara Health Institute, Health Systems, Impact Evaluation and Policy<br>Mashasi, Irene; Ifakara Health Institute, Health Systems, Impact Evaluation and Policy<br>Karugu, Caroline; African Population and Health Research Center, Chronic Disease Management Unit<br>Mohamed, Shukri; African Population and Health Research Center, Chronic Disease Management Unit<br>Asiki, G; African Population and Health Research Center, Chronic Disease Management Unit<br>Mair, Frances; University of Glasgow, School of Health and Wellbeing<br>Gray, Cindy; University of Glasgow, School of Social and Political Sciences; University of Glasgow, School of Health and Wellbeing |
| <b>Primary Subject Heading</b>: | Public health |
| Secondary Subject Heading: | Diabetes and endocrinology, Health policy, Health services research, Public health, Qualitative research |
| Keywords: | COVID-19, Health policy < HEALTH SERVICES ADMINISTRATION & MANAGEMENT, Public health < INFECTIOUS DISEASES, Health Services Accessibility |
| | |

SCHOLARONE™
Manuscripts

*I, the Submitting Author has the right to grant and does grant on behalf of all authors of the Work (as defined in the below author licence), an exclusive licence and/or a non-exclusive licence for contributions from authors who are: i) UK Crown employees; ii) where BMJ has agreed a CC-BY licence shall apply, and/or iii) in accordance with the terms applicable for US Federal Government officers or employees acting as part of their official duties; on a worldwide, perpetual, irrevocable, royalty-free basis to BMJ Publishing Group Ltd ("BMJ") its licensees and where the relevant Journal is co-owned by BMJ to the co-owners of the Journal, to publish the Work in this journal and any other BMJ products and to exploit all rights, as set out in our licence.*

*The Submitting Author accepts and understands that any supply made under these terms is made by BMJ to the Submitting Author unless you are acting as an employee on behalf of your employer or a postgraduate student of an affiliated institution which is paying any applicable article publishing charge ("APC") for Open Access articles. Where the Submitting Author wishes to make the Work available on an Open Access basis (and intends to pay the relevant APC), the terms of reuse of such Open Access shall be governed by a Creative Commons licence – details of these licences and which Creative Commons licence will apply to this Work are set out in our licence referred to above.*

*Other than as permitted in any relevant BMJ Author's Self Archiving Policies, I confirm this Work has not been accepted for publication elsewhere, is not being considered for publication elsewhere and does not duplicate material already published. I confirm all authors consent to publication of this Work and authorise the granting of this licence.*

For peer review only

# Factors associated with COVID-19 vaccine uptake among people with type 2 diabetes in Kenya and Tanzania: a mixed-methods study

Peter Binyaruka[1#*], Sally Mtenga[1#], Irene Mashasi[1], Caroline Karugu[2], Shukri F. Mohamed[2], Gershim Asiki[2], Frances S. Mair[3], Cindy M. Gray[3,4]

**Author affiliations**

[1] Department of Health system, Impact Evaluation, and Policy, Ifakara Health Institute, PO Box 78373, Dar es Salaam, Tanzania.

[2] Chronic Disease Management Unit, African Population and Health Research Center (APHRC), Nairobi, Kenya.

[3] School of Health and Wellbeing, University of Glasgow, Glasgow, UK

[4] School of Social and Political Sciences, University of Glasgow, Glasgow, UK

* **Corresponding author:** Peter Binyaruka, *pbinyaruka@ihi.or.tz, +255 (0) 655 363361*

[#] **Joint first authors:** Peter Binyaruka, Sally Mtenga

**E-mail addresses:**

Peter Binyaruka: pbinyaruka@ihi.or.tz

Sally Mtenga: smtenga@ihi.or.tz

Irene Mashasi: imashasi@gmail.com

Caroline Karugu: ckarugu@aphrc.org

Shukri F. Mohamed: smohamed@aphrc.org

Gershim Asiki: gasiki@aphrc.org

Frances S. Mair: Frances.Mair@glasgow.ac.uk

Cindy M. Gray: Cindy.Gray@glasgow.ac.uk

**Word Count:** 3439 words

**Key words:** COVID-19, vaccine uptake, determinants, barriers, facilitators, hesitancy, Tanzania.

For peer review only

**Abstract (250 words)**

**Background:** People with type 2 diabetes (T2D) are at increased risk of poor outcomes from COVID-19. Vaccination can improve outcomes, but vaccine hesitancy remains a major challenge. We examined factors influencing COVID-19 vaccine uptake among people with T2D in two sub-Saharan Africa countries that adopted different national approaches to combat COVID-19, Kenya and Tanzania.

**Methods:** A mixed-methods study, involving a survey of 1000 adults with T2D (500 Kenya; 500 Tanzania) and 51 in-depth interviews (21 Kenya; 30 Tanzania). Determinants of COVID-19 vaccine uptake were identified using multivariate logistic regression, while thematic content analysis explored barriers and facilitators.

**Results:** COVID-19 vaccine uptake was lower in Tanzania (26%) than in Kenya (75%), which may reflect an initial political hesitancy about vaccines in Tanzania. People with college/university education were four times more likely to be vaccinated than those with no education (Kenya AOR=4.25 [95%CI:1.00-18.03], Tanzania AOR=4.07 [1.03-16.12]); and people with health insurance were almost twice as likely to be vaccinated than those without health insurance (Kenya AOR=1.70 [1.07-2.70], Tanzania AOR=1.81 [1.04-3.13]). Vaccine uptake was higher in older people in Kenya, and among those with more comorbidities and higher socioeconomic status in Tanzania. Interviewees reported that wanting protection from severe illness promoted vaccine uptake, while conflicting information, misinformation and fear of side-effects limited uptake.

**Conclusion:** COVID-19 vaccine uptake among people with T2D was suboptimal, particularly in Tanzania, where initial political hesitancy had a negative impact. Policymakers must develop strategies to reduce fear and misconceptions, especially among those who are less educated, uninsured, and younger.

For peer review only

**Strengths and limitations of this study**

- This is the first large population-based study to examine COVID-19 vaccine uptake among people with T2D in two low-and middle-income countries in sub-Saharan Africa.
- The design allowed an exploration of the factors, including political factors, that influenced vaccine uptake in two countries with different approaches to combatting COVID-19.
- The mixed-methods study design provided in-depth contextual insights of people's attitudes to and experiences of COVID-19 vaccination.
- A limitation was that COVID-19 vaccination status was based on self-report rather than clinical records.

**INTRODUCTION (3439 words)**

The COVID-19 pandemic has negatively impacted social, health and economic welfare globally. In the health sector, COVID-19 led to healthcare disruptions affecting routine care, especially for patients living with chronic or non-communicable diseases (NCDs) like type 2 diabetes (T2D)[1]. People with T2D have a compromised immune system and are at higher risk of infection and adverse outcomes from COVID-19 [2, 3].

Vaccination is a proven pharmaceutical intervention to reduce COVID-19 transmission and adverse outcomes [4]. In December 2020, newly developed COVID-19 vaccines were authorised, and efforts by the COVID-19 Vaccines Global Access (COVAX) initiative aimed to ensure global vaccine equity. However, vaccination coverage is still suboptimal in many low-and middle-income countries (LMICs) due to limited COVAX vaccine supply [5] and vaccine hesitancy because of concerns about life-threatening side effects, risk of new diseases, and infertility [6-10].

High vaccine coverage is required to reduce incidence of COVID-19 and hospitalisation. In many countries, people with chronic diseases, such as T2D, are prioritised during national

COVID-19 vaccine rollouts [11]. It is therefore important for policymakers to understand the prevalence of COVID-19 vaccine uptake and factors influencing uptake among people with chronic diseases. A few studies among people with T2D, including only two from sub-Saharan Africa, revealed that vaccine uptake was significantly higher among men, public servants, urban populations and people with a higher education level [12-20]. This paper extends the evidence base by estimating the prevalence of COVID-19 vaccine uptake among people with T2D in Kenya and Tanzania, and identifying the factors influencing uptake in both countries. We chose these two countries because they took different approaches to combat COVID-19 (e.g., 2020 lockdown in Kenya, but not in Tanzania; initial government hesitancy about COVID 19 vaccines in Tanzania, but not in Kenya). We focused on T2D because it is a risk factor for other chronic illness such as kidney failure, cardiovascular disease and hypertension [21], and increases vulnerability and severity of COVID-19 [2, 3]. Our findings will guide targeted communication campaigns and other strategies to improve vaccination coverage, especially among people with chronic diseases, such as T2D.

For peer review only

## METHODS

### Study setting

This study was conducted in two LMICs in East Africa, Kenya and Tanzania. We purposively selected four counties (two urban – Nairobi, Kiambu; two rural – Vihiga, Nyeri) out of 47 counties in Kenya, and two regions (one urban – Dar es Salaam; one rural – Morogoro) out of 31 regions in Tanzania. The prevalence of diabetes mellitus was relatively lower in Kenya (2.4% in 2015)[22] than in Tanzania (9.1% in 2012) [23]. By August 2023, Tanzania and Kenya had recorded 43,078 and 343,918 confirmed cases and 846 and 5,689 COVID-19-related deaths, respectively [24]. COVID-19 vaccination is free in both countries, but priority was initially given to health workers, older and people with chronic health conditions. Both countries received vaccines through COVAX, but the vaccination rollout began slightly earlier in Kenya (March 2021)[25] compared to Tanzania (July 2021) [26].

### Study design


For peer review only - http://bmjopen.bmj.com/site/about/guidelines.xhtml

We use a mixed-methods study design involving a cross-sectional survey of 500 people with T2D in each country, and qualitative in-depth interviews with 21 and 30 people with T2D in Kenya and Tanzania, respectively.

**Study participants and sampling**

The survey included adults (18+ years) who were diagnosed with T2D before COVID-19 (i.e., before March 2020) in Nairobi (n=276), Kiambu (n=104), Vihiga (n=76), and Nyeri (n=44) counties in Kenya, and in Dar es Salaam (n=300) and Morogoro (n=200) regions in Tanzania. The sample size of 500 people was estimated using the Cochran formula, assuming 50% of patients with T2D experienced disruption of care during COVID-19[27], with 80% power, a 5% margin of error and a 30% non-response rate. Adult patients with T2D who received care in selected hospitals and health centres before March 2020 were identified from health facility outpatient registers and approached for written informed consent. Based on convenience sampling, participants for the in-depth interviews were mainly selected from survey participants. In both countries, data collection stopped once saturation of views was achieved. People with T2D who were vaccinated or unvaccinated, and with or without other health conditions were eligible to take part in both the survey and in-depth interviews.

**Data collection and recruitment**

Fieldworkers were recruited from each country and trained for four days before data collection. Trained fieldworkers administered structured survey questionnaires in both countries and used an interview topic guide (Supplementary file 1) to explore facilitators and barriers to COVID-19 vaccination. Both tools were piloted and refined before use. Data collection was conducted in February-March 2022. In Tanzania, all data collection was conducted face-to-face at the health facility in a room or other location that ensured full privacy for the participant. In Kenya, the survey was administered by phone due to national guidelines to minimise COVID-19 transmission, while the decision was taken to conduct the qualitative interviews face-to-face as they involved a much smaller sample size, and thus lower risk. Data collection was conducted in Swahili in Tanzania, while in Kenya, where fieldworkers were fluent in Swahili and English, participants were allowed to respond in either language.

**Measures**


For peer review only - http://bmjopen.bmj.com/site/about/guidelines.xhtml

Our primary outcome of interest was COVID-19 vaccine uptake among people with T2D in Kenya and Tanzania and was assessed using the following question: *'Have you been vaccinated for COVID-19?'*. We used a binary outcome with a value of 1 if a participant confirmed having received any COVID-19 vaccine (regardless of dosage) by February 2022, and 0 otherwise.

The explanatory variables as potential determinants of COVID-19 vaccine uptake were based on the World Health Organization's (WHO) social determinants of health conceptual framework[28] and relevant empirical evidence on factors influencing health care seeking behaviour, particularly vaccination [29]. We used two categories of determinants: socio-demographic and health-related.

*Socio-demographic factors* included five binary variables: place of residence (rural/urban), sex (male/female), marital status (married/not married), health insurance (insured/non-insured), and socioeconomic status (SES) based on a ladder scale (1-10) that asked participants to rank their households in terms of economic position, where 1 was lowest economic position and 10 was highest economic position (lower 1-5/higher 6-10). We also included four categorical variables: age (<40/40-49/50-59/60-69/>70 years), education level (no education/primary/secondary/higher education), religion (Catholic/Protestant/Muslim), and occupation status (formal sector workers/farmers/self-employed/retired/unemployed).

*Health-related factors* included three binary variables: family history of T2D (history/no history), time living with T2D (<6 years/≥6 years), and presence of comorbidities (with/without comorbidities). The number of comorbidities was assessed by asking participants to report additional long-term conditions.

**Data analysis**

Descriptive analysis was undertaken to describe participants' socio-demographic and health-related characteristics and prevalence of COVID-19 vaccination in each country. To identify the independent determinants of vaccine uptake, we performed separate multivariate logistic regressions for each country. Since vaccine uptake was higher in Kenya (75% - reflecting a non-rare event), we checked the sensitivity of the logistic regression by applying a modified Poisson regression model [30]. We assessed multicollinearity between independent variables using a pairwise correlation analysis. All correlation coefficients were below 0·5, which is



For peer review only - http://bmjopen.bmj.com/site/about/guidelines.xhtml

considered moderate, supporting the inclusion of all variables in the regression analysis. STATA version 16 was used to analyse the quantitative data.

Audio-recorded data from the qualitative interviews were transcribed verbatim in Kenya and Tanzania. Researchers repeatedly reviewed the transcripts to familiarize themselves with the data. Thematic content analysis was employed to identify common phrases related to the factors that facilitated and deterred COVID-19 vaccine uptake. In this process, inductive coding was used independently by researchers from each study country to categorise the key themes. They then met to discuss and agree on the codes that were applied to all transcripts. NVivo software (version 10 in Kenya, version 12 Tanzania) was used to manage and analyse the data.

**Patient and public involvement:** Patients and/or the public were not involved in the design, or conduct, or reporting, or dissemination plans of this research.

## RESULTS

### Participant characteristics

Most participants in both countries were from urban settings (>59%), female (>65%), and married (>62%) (Table 1). The majority were also aged above 50 years (the average age was 58 years in Kenya and 57 years in Tanzania) and had completed primary or secondary education. However, religious affiliation differed between the two countries, with almost equal numbers of Muslims and Christians in Tanzania, while Christians dominated in Kenya. In both countries, the majority of participants worked as farmers or were self-employed, had health insurance (>60%), and were of lower SES (89% in Kenya, 75% in Tanzania). Fewer participants in Tanzania (42%) had a family history of T2D than in Kenya (53%). Most patients in both countries had lived with T2D for 6 years or more (>61%), while the mean number of comorbidities was 3.4 in Kenya and 1.5 in Tanzania.

Most people who participated in the qualitative in-depth interviews had comorbidities (particularly hypertension). They were aged between 60 and 80 years in Kenya, and between 46 and 51 years in Tanzania. In urban areas, interviewees were employed/running businesses, retired or unemployed, while in rural areas the majority were farmers.


For peer review only - http://bmjopen.bmj.com/site/about/guidelines.xhtml

**Factors influencing COVID-19 vaccine uptake**

The survey data showed that COVID-19 vaccine uptake was relatively lower in Tanzania than in Kenya. Only 26% of participants in Tanzania reported having received at least one COVID-19 vaccination, compared with 75% in Kenya. The qualitative interviews suggested that the low rate of vaccine uptake in Tanzania may reflect the political approach to COVID-19 in that country. Initially, the past presidential regime promoted natural remedies like lemon, ginger, and steam with herbal remedies to combat COVID-19, as well as WHO recommended prevention measures (e.g., masks and hands sanitizer), but did not endorse COVID-19 vaccination. Despite the current Tanzanian government's support and promotion of COVID-19 vaccination rollout from July 2021, the initial political hesitancy emerged as a continued barrier to vaccine uptake in the accounts of Tanzanian interviewees.

> *"I cannot say the COVID-19 vaccine is good because our late President said the vaccine cannot protect us from COVID-19 rather we should protect ourselves and said absolutely there is no vaccine to cure COVID-19, so we are following him"* **[Female-PT09-urban-Tanzania]**.

The survey data showed that participants with a college/university education were over four times more likely to be vaccinated than those with no education in Kenya (AOR=4.25, 95%CI: 1.00-18.03, $p<0.05$) and Tanzania (AOR=4.07, 95%CI: 1.03-16.12, $p<0.05$) (Table 2). The qualitative interviews revealed the importance of education, particularly in terms of understanding and weighing up the complex information available about COVID-19. For instance, some patients who were vaccinated in both countries felt that protecting themselves from the risk of severe illness outweighed any perceived risk from the COVID-19 vaccine.

> *"I just think it is good. I took AstraZeneca and it did not affect me in any way. In fact, it gave me a sense of safety. You feel you are a little bit safe"* **[Female-220312_1439-rural-Kenya]**

However, some interviewees also described the difficulty people faced in trying to navigate the diverse and sometimes conflicting information from different sources, such as government leaders, religious leaders, and health care professionals. Some participants reported using wider evidence, such as the international response to COVID-19, to inform their decision to get vaccinated:



For peer review only - http://bmjopen.bmj.com/site/about/guidelines.xhtml

> *"I feel like what the government is saying, other countries are also doing it, I don't see how it can be a bad thing"* **[Female-220312_1439-rural-Kenya].**

In addition to formal sources of information, people in both countries also used informal local knowledge, which was not always helpful, to inform their decision-making. Interviewees, particularly in rural Tanzania, described how myths and misconceptions about the COVID-19 vaccine in their communities increased vaccine hesitancy locally:

> *"People say if you vaccinate you can become vampire-like and later you will be like a zombie! You can even give birth to an abnormal child"* **[Female-PT12-rural Tanzania].**

These myths and misconceptions were fuelled by the speed of the development and rollout of COVID-19 vaccines. Interviewees in both countries, expressed concerns about vaccine quality and safety, including the potential of adverse side effects. For, example one man from rural Kenya described how the fear was intensified for many people with T2D:

> *"I think even now most diabetic people are afraid of getting the vaccine. I was also afraid at first because it was believed that if you get vaccinated and you have diabetes you will die"* **[Male-220312_1220-rural-Kenya].**

Health insurance emerged as another strong predictor of vaccine uptake in the quantitative analysis: people with health insurance were almost twice as likely to be vaccinated than those who were not insured in Kenya (AOR=1.70, 95%CI: 1.07-2.70, $p<0.05$) and Tanzania (AOR=1.81, 95%CI: 1.04-3.13, $p<0.05$) (Table 2). The interviews suggested that the lower vaccine uptake among participants without health insurance may in part reflect the fact that the financial hardship caused by the pandemic meant that accessing healthcare for those who were not insured was less important than other competing priorities:

> *"I run a small business, so, whatever I get I put it aside to feed my children, and some to buy my diabetic treatment because I don't have health insurance. If I had the insurance, it would have been easier, I could just come to the hospital and I will be given medication. But now I need to buy (medicine) and the economic situation isn't good with this CORONA at hand. If I had the money, I would buy medicines to take me for two weeks or a month, and I would not miss any dose, but now I can't"* [**Female PT03-urban-Tanzania**].


For peer review only - http://bmjopen.bmj.com/site/about/guidelines.xhtml

In Kenya, older patients (50-59 years and 60-69 years) had a higher odds of vaccine uptake (AOR=2.51, 95%CI: 1.10-5.72, $p<0.05$) and (AOR=2.48, 95%CI: 1.05-5.84, $p<0.05$), respectively, compared to young patients (<40 years) (Table 2). In Tanzania (but not Kenya), having more comorbidities was associated with higher vaccine uptake (AOR=1.28, 1.01-1.61, $p<0.05$). Being married showed a weak positive association with vaccine uptake in both countries ($p<0.10$), and lower socioeconomic status was weakly associated with lower vaccine uptake in Tanzania only ($p=0.059$). The Modified Poisson regression results (Appendix Table 1) were broadly similar to the logistic regression results in terms of associations and levels of statistical significance of both odds ratios and risk ratios. However, the associations between higher education and being married and vaccine uptake disappeared in Kenya. It is important to note that the results between two models are not directly comparable because the logistic model used odds ratio while Modified Poisson model used risk ratios.

## DISCUSSION

Our investigation of COVID-19 vaccine uptake among people with T2D found that 75% and 26% reported having had at least one vaccination in Kenya and Tanzania, respectively. Vaccine uptake was lower in Tanzania partly because of the vaccine hesitancy of the past presidential regime by arguing to conduct a robust evaluation before accepting the use of vaccines [31-33], which continued to influence people's decision making around whether to be vaccinated, despite the subsequent government's promotion of vaccination during the national rollout from July 2021 [26]. Being better educated and having health insurance were significantly associated with higher COVID-19 vaccine uptake in both countries. Vaccine uptake was also higher among older people with T2D in Kenya, and among those with increased number of comorbidities in Tanzania. Interviewees reported that concerns about becoming severely ill if they contracted COVID-19 informed their decision to get vaccinated, while fear of vaccine side effects/safety/quality, different and sometimes conflicting information on vaccines, and local myths and misinformation limited vaccine uptake.

The finding that higher education levels were associated with increased vaccine uptake in both countries (although in Kenya the association disappeared in the Poisson sensitivity analysis) is consistent with previous research on COVID-19 vaccination among people with chronic illness including diabetes in Australia [34] and Italy [13], and with a study showing an association between better education and intention to get the COVID-19 vaccine among chronically ill


For peer review only - http://bmjopen.bmj.com/site/about/guidelines.xhtml

people in Saudi Arabia [15]. However, a similar study in Sudan showed no association between education and vaccine uptake [9].

The finding that having health insurance was associated with increased COVID-19 vaccine uptake among people with T2D in both countries is consistent with the results of a study of people with chronic conditions who had health insurance in Ethiopia [16]. Evidence suggests that having health insurance improves people's access to health care services [35], which may help people with health insurance feel more empowered to access vaccines than those without health insurance.

The fact that in Tanzania, people with more comorbidities were more likely to have been vaccinated likely reflects their perception of increased vulnerability from contracting COVID-19. This interpretation of the quantitative findings is supported by the qualitative accounts that COVID-19 vaccination was thought to offer protection from severe illness. The findings may also reflect the success of the Tanzanian government's strategy to sensitise and prioritise vulnerable population groups during the July 2021 COVID-19 vaccine rollout. However, the number of comorbidities was not associated with COVID-19 vaccine uptake in Kenya, which may reflect the higher overall vaccination coverage.

In our study, being older was associated with higher COVID-19 vaccine uptake in Kenya, albeit a weaker association in the Poisson sensitivity analysis. This  finding is consistent with a study conducted across five sub-Saharan African countries (Burkina Faso, Ethiopia, Ghana, Nigeria, and Tanzania), which reported that vaccine hesitancy was extremely high among young people (in this case adolescents); including 14% in rural Kersa, 23% in rural Ibadan, 31% in rural Nouna, 32% in urban Ouagadougou, 37% in urban Addis Ababa, 48% in rural Kintampo, 65% in urban Lagos, 76% in urban Dar es Salaam, and 88% in rural Dodoma [36]. However, other studies have shown mixed associations between vaccine uptake and age among people with T2D. For instance, age was positively associated with COVID-19 vaccination in Australia [34], but negatively associated in Saudi Arabia [37]. Higher SES was also weakly associated with increased vaccine uptake in Tanzania, which supports the results of previous studies that included people with T2D in Australia [34], China [8] and Ethiopia [16].

The fear of vaccine side effects, safety, and quality emerged among factors limiting the uptake of COVID-19 vaccination in both countries. These findings are consistent with other literature about COVID-19 vaccine hesitancy in LMICs [6-10, 33], which have highlighted particular



For peer review only - http://bmjopen.bmj.com/site/about/guidelines.xhtml

concerns about death, developing new diseases, and infertility. Such concerns have been heightened by the rapid development and production of COVID-19 vaccines and the novel mRNA-based vaccine technology [38].

To our knowledge this is the first large population-based, mixed-methods study to examine COVID-19 vaccine uptake, as well as the factors influencing uptake, among people with T2D in two LMICs with different national approaches to combatting COVID-19. The study offers insights into the impact on vaccine uptake of these different approaches, which included initial political hesitancy towards vaccination in Tanzania which delayed the vaccination rollout, compared to a pro-vaccine view within the Kenyan government. It is also the first study to examine the influence of comorbidities on COVID-19 vaccine uptake in people with T2D.

Our study had some limitations. First, we were unable to get a fully representative sample of people with T2D in each country, because we only recruited those who were diagnosed and had attended care in health facilities. Vaccine uptake may be higher in this sub-group because they are more engaged with health services than those who do not attend local health facilities. Future studies should recruit people with T2D in community settings to include those who are undiagnosed and/or not registered at health facilities. Second, our study assessed vaccine uptake when national rollout had not yet been fully implemented, particularly in Tanzania. However, in both Kenya and Tanzania, people with chronic diseases such as T2D were prioritized, so it is likely that our study participants would all have been offered at least one vaccination. Third, as COVID-19 vaccination status was self-reported, social desirability bias may have inflated reports of being vaccinated. Fourth, we were unable to use the past history of COVID-19 infection as a predictor of COVID-19 vaccine uptake, because our data had few participants who confirmed to have contracted COVID-19 infection (e.g., only 4% of patients with T2D confirmed to have contracted COVID-19 infection in Tanzania).

Our study has important implications for improving COVID-19 vaccine uptake among people with chronic diseases like T2D in countries like Kenya and Tanzania. It highlights the need for governments to engage with and promote evidence-based health advice (such as the effectiveness of COVID-19 vaccines in reducing disease transmission and severity) to encourage vaccine uptake. It also provides a basis to help policymakers in Kenya and Tanzania develop clear policies and strategies to reduce vaccine hesitancy among people with T2D and other chronic diseases. Potential strategies for consideration include national COVID-19 vaccination education campaigns targeting different 'vaccine hesitant' sub-groups, such as



For peer review only - http://bmjopen.bmj.com/site/about/guidelines.xhtml

those who are less well educated (including low SES), those who do not have health insurance and younger people. However, it is also important to counter the anti-vaccination views, perceptions and beliefs that can rapidly undermine efforts to promote vaccine uptake [39], for example, by using influential leaders and role models in local and national multi-media communication campaigns.

## Ethics

Ethical approval was obtained from respective ethics committees in Kenya and Tanzania. In Kenya, the permit was obtained from the African Population and Health Research Center Ethics Review Committee (ref. no: DOR 2021/041), African Medical and Research Foundation (AMREF) Health Africa's Ethics and Scientific Research Committee (ref. no: AMREF-ESRC P900/2020), and National Commission for Science, Technology and Innovation (licence no: NACOSTI/p22/14986). In Tanzania, the institutional ethical approval was from the Ifakara Health Institute (IHI/IRB/No: 38-2021), while the national approval was from the National Institute of Medical Research (NIMR/HQ/R.8a/Vol.X/3806). We sought written informed consent from all respondents by providing information on the objectives of the research and the procedures applied in a clear language using an information sheet.

**Conflict of interest:** None.

**Authors' contributions:** SM, GA, FM and CMG designed the study. PB and CK analyzed the survey data with support from GA. IM, SM and SFM analyzed qualitative data from both countries. SM, CMG, FM and GA contributed to data interpretation. PB and SM wrote jointly the first draft of the manuscript. CMG, FM and GA supported development of subsequent drafts.  All authors approved the final manuscript.

**Funding:** Medical Research Council (MRC) and the National Institute for Health Research (NIHR) [MR/V035924/1].

**Data availability statement:** Data is available on request. There are ethical restrictions on sharing the data set publicly, one reason being data containing sensitive patient information particularly the experience with COVID-19. The restriction was imposed by the Ifakara Health Institute (IHI) Institutional Review Board (IRB). In case of data request, please contact the IRB secretary, Dr. Mwifadhi Mrisho (+255655766675, mmrisho@ihi.or.tz).



For peer review only - http://bmjopen.bmj.com/site/about/guidelines.xhtml

**Patient consent:** Obtained.

## REFERENCES (39 ref)


1. Chang AY, Cullen MR, Harrington RA, Barry M: **The impact of novel coronavirus COVID-19 on noncommunicable disease patients and health systems: a review**. *Journal of internal medicine* 2021, **289**(4):450-462.
2. Fang L, Karakiulakis G, Roth M: **Are patients with hypertension and diabetes mellitus at increased risk for COVID-19 infection?** *Lancet Respir Med* 2020, **8**(4):e21.
3. Huang I, Lim MA, Pranata R: **Diabetes mellitus is associated with increased mortality and severity of disease in COVID-19 pneumonia - A systematic review, meta-analysis, and meta-regression**. *Diabetes Metab Syndr* 2020, **14**(4):395-403.
4. Watson OJ, Barnsley G, Toor J, Hogan AB, Winskill P, Ghani AC: **Global impact of the first year of COVID-19 vaccination: a mathematical modelling study**. *The Lancet Infectious Diseases* 2022.
5. Wouters OJ, Shadlen KC, Salcher-Konrad M, Pollard AJ, Larson HJ, Teerawattananon Y, Jit M: **Challenges in ensuring global access to COVID-19 vaccines: production, affordability, allocation, and deployment**. *The Lancet* 2021, **397**(10278):1023-1034.
6. Piltch-Loeb R, Harriman NW, Healey J, Bonetti M, Toffolutti V, Testa MA, Su M, Savoia E: **COVID-19 Vaccine Concerns about Safety, Effectiveness, and Policies in the United States, Canada, Sweden, and Italy among Unvaccinated Individuals**. *Vaccines (Basel)* 2021, **9**(10):1138.
7. Diaz P, Zizzo J, Balaji NC, Reddy R, Khodamoradi K, Ory J, Ramasamy R: **Fear about adverse effect on fertility is a major cause of COVID-19 vaccine hesitancy in the United States**. *Andrologia* 2022, **54**(4):e14361.
8. Duan L, Wang Y, Dong H, Song C, Zheng J, Li J, Li M, Wang J, Yang J, Xu J: **The COVID-19 Vaccination Behavior and Correlates in Diabetic Patients: A Health Belief Model Theory-Based Cross-Sectional Study in China, 2021**. *Vaccines (Basel)* 2022, **10**(5).
9. Omar SM, Khalil R, Adam I, Al-Wutayd O: **The Concern of COVID-19 Vaccine Safety Is behind Its Low Uptake among Patients with Diabetes Mellitus in Sudan**. *Vaccines (Basel)* 2022, **10**(4).
10. Kabakama S, Konje ET, Dinga JN, Kishamawe C, Morhason-Bello I, Hayombe P, Adeyemi O, Chimuka E, Lumu I, Amuasi J: **Commentary on COVID-19 Vaccine Hesitancy in sub-Saharan Africa**. *Tropical Medicine and Infectious Disease* 2022, **7**(7):130.
11. Liu Y, Sandmann FG, Barnard RC, Pearson CA, Pastore R, Pebody R, Flasche S, Jit M: **Optimising health and economic impacts of COVID-19 vaccine prioritisation strategies in the WHO European Region: a mathematical modelling study**. *The Lancet Regional Health-Europe* 2022, **12**:100267.


For peer review only

12. Aldossari KK, Alharbi MB, Alkahtani SM, Alrowaily TZ, Alshaikhi AM, Twair AA: **COVID-19 vaccine hesitancy among patients with diabetes in Saudi Arabia**. *Diabetes Metab Syndr* 2021, **15**(5):102271.
13. Guaraldi F, Montalti M, Di Valerio Z, Mannucci E, Nreu B, Monami M, Gori D: **Rate and Predictors of Hesitancy toward SARS-CoV-2 Vaccine among Type 2 Diabetic Patients: Results from an Italian Survey**. *Vaccines (Basel)* 2021, **9**(5).
14. Asadi-Pooya AA, Barzegar Z, Sadeghian S, Nezafat A, Shahisavandi M, Nabavizadeh SA: **COVID-19 Vaccine Hesitancy Among Patients With Epilepsy or Other Chronic Conditions**. *Disaster Med Public Health Prep* 2021:1-3.
15. Al-Hanawi MK, Ahmad K, Haque R, Keramat SA: **Willingness to receive COVID-19 vaccination among adults with chronic diseases in the Kingdom of Saudi Arabia**. *J Infect Public Health* 2021, **14**(10):1489-1496.
16. Kassa Mekonnen C, Gizaw Demissie N, Wako Beko Z, Mulu Ferede Y, Kindie Abate H: **Intent to get vaccinated against COVID-19 pandemic and its associated factors among adults with a chronic medical condition**. *Int J Afr Nurs Sci* 2022, **16**:100401.
17. Kolobov T, Djuraev S, Promislow S, Tamir O: **Determinants of COVID-19 vaccine acceptance among adults with diabetes and in the general population in Israel: A cross-sectional study**. *Diabetes Res Clin Pract* 2022, **189**:109959.
18. Osuagwu UL, Langsi R, Ovenseri-Ogbomo G, Mashige KP, Abu EK, Envuladu EA, Goson PC, Ekpenyong BN, Oloruntoba R, Miner CA *et al*: **Analysis of Perception, Reasons, and Motivations for COVID-19 Vaccination in People with Diabetes across Sub-Saharan Africa: A Mixed-Method Approach**. *Int J Environ Res Public Health* 2022, **19**(13).
19. Scoccimarro D, Panichi L, Ragghianti B, Silverii A, Mannucci E, Monami M: **Sars-CoV2 vaccine hesitancy in Italy: A survey on subjects with diabetes**. *Nutr Metab Cardiovasc Dis* 2021, **31**(11):3243-3246.
20. Wang Y, Duan L, Li M, Wang J, Yang J, Song C, Li J, Wang J, Jia J, Xu J: **COVID-19 Vaccine Hesitancy and Associated Factors among Diabetes Patients: A Cross-Sectional Survey in Changzhi, Shanxi, China**. *Vaccines (Basel)* 2022, **10**(1).
21. WHO: **Diabetes Fact Sheet**. In.: World Health Organization; 2022.
22. Mohamed SF, Mwangi M, Mutua MK, Kibachio J, Hussein A, Ndegwa Z, Owondo S, Asiki G, Kyobutungi C: **Prevalence and factors associated with pre-diabetes and diabetes mellitus in Kenya: results from a national survey**. *BMC Public Health* 2018, **18**(3):1215.
23. Mayige M, Kagaruki G, Ramaiya K, Swai A: **Non communicable diseases in Tanzania: a call for urgent action**. *Tanzan J Health Res* 2011, **13**(5 Suppl 1):378-386.
24. **WHO Health Emergency Dashboard** [https://covid19.who.int]
25. Orangi S, Pinchoff J, Mwanga D, Abuya T, Hamaluba M, Warimwe G, Austrian K, Barasa E: **Assessing the level and determinants of COVID-19 vaccine confidence in Kenya**. *Vaccines (Basel)* 2021, **9**(8):936.
26. **The United Republic of Tanzania receives the first COVAX shipment**
27. WHO: **The impact of the COVID-19 pandemic on noncommunicable disease resources and services: results of a rapid assessment**. In.: World Health Organization (WHO), Geneva, Switzerland; 2020.
28. Solar O, Irwin A: **A conceptual framework for action on the social determinants of health. Social Determinants of Health**. In: *Discussion Paper 2 (Policy and Practice).* 2010.




For peer review only - http://bmjopen.bmj.com/site/about/guidelines.xhtml

29. Phillips DE, Dieleman JL, Lim SS, Shearer J: **Determinants of effective vaccine coverage in low and middle-income countries: a systematic review and interpretive synthesis**. *BMC Health Services Research* 2017, **17**(1):681.
30. Zou G: **A modified poisson regression approach to prospective studies with binary data**. *Am J Epidemiol* 2004, **159**(7):702-706.
31. Makoni M: **Tanzania refuses COVID-19 vaccines**. *The Lancet* 2021, **397**(10274):566.
32. Mfinanga SG, Mnyambwa NP, Minja DT, Ntinginya NE, Ngadaya E, Makani J, Makubi AN: **Tanzania's position on the COVID-19 pandemic**. *The Lancet* 2021, **397**(10284):1542-1543.
33. Mtenga S, Mhalu G, Osetinsky B, Ramaiya K, Kassim T, Hooley B, Tediosi F: **Social-political and vaccine related determinants of COVID-19 vaccine hesitancy in Tanzania: A qualitative inquiry**. *PLOS Glob Public Health* 2023, **3**(6):e0002010.
34. Day D, Grech L, Nguyen M, Bain N, Kwok A, Harris S, Chau H, Chan B, Blennerhassett R, Nott L *et al*: **Serious Underlying Medical Conditions and COVID-19 Vaccine Hesitancy: A Large Cross-Sectional Analysis from Australia**. *Vaccines (Basel)* 2022, **10**(6).
35. Amani PJ, Hurtig AK, Frumence G, Kiwara AD, Goicolea I, San Sebastiån M: **Health insurance and health system (un) responsiveness: a qualitative study with elderly in rural Tanzania**. *BMC Health Serv Res* 2021, **21**(1):1140.
36. Wang D, Chukwu A, Mwanyika-Sando M, Abubakari SW, Assefa N, Madzorera I, Hemler EC, Ismail A, Lankoande B, Mapendo F *et al*: **COVID-19 vaccine hesitancy and its determinants among sub-Saharan African adolescents**. *PLOS Global Public Health* 2022, **2**(10):e0000611.
37. Tourkmani AM, Bin Rsheed AM, AlEissa MS, Alqahtani SM, AlOtaibi AF, Almujil MS, IH AL, TN AL, MN AL, Alrasheedy AA: **Prevalence of COVID-19 Infection among Patients with Diabetes and Their Vaccination Coverage Status in Saudi Arabia: A Cross-Sectional Analysis from a Hospital-Based Diabetes Registry**. *Vaccines (Basel)* 2022, **10**(2).
38. van Riel D, de Wit E: **Next-generation vaccine platforms for COVID-19**. *Nature materials* 2020, **19**(8):810-812.
39. Prieto Curiel R, González Ramírez H: **Vaccination strategies against COVID-19 and the diffusion of anti-vaccination views**. *Scientific Reports* 2021, **11**(1):1-13.

For peer review only

List of Tables

**Table 1: Socio-demographic and health-related characteristics of T2D patients**

| Variable | Description | Kenya (N=500) | | Tanzania (N=500) | |
|---|---|---|---|---|---|
| | | n | % | n | % |
| Place of residence | Urban | 380 | 76.0% | 300 | 60.0% |
| | Rural | 120 | 24.0% | 200 | 40.0% |
| Sex | Female | 330 | 66.0% | 336 | 67.2% |
| | Male | 170 | 34.0% | 164 | 32.8% |
| Marital status | Married | 319 | 63.8% | 312 | 62.4% |
| | Not married | 181 | 36.2% | 188 | 37.6% |
| Age group | <40 years | 39 | 7.8% | 36 | 7.2% |
| | 40-49 years | 80 | 16.0% | 62 | 12.4% |
| | 50-59 years | 135 | 27.0% | 165 | 33.0% |
| | 60-69 years | 155 | 31.0% | 206 | 41.2% |
| | ≥70 years | 91 | 18.2% | 31 | 6.2% |
| Mean age in years [SD] | | 500 | 58.2 [12.6] | 500 | 56.8 [10.2] |
| Education level | No education | 16 | 3.2% | 34 | 6.8% |
| | Primary education | 208 | 41.6% | 298 | 59.6% |
| | Secondary education | 201 | 40.2% | 126 | 25.2% |
| | Higher education | 75 | 15.0% | 42 | 8.4% |

For peer review only

| | | | | | |
|---|---|---|---|---|---|
| **Religion** | Catholic | 104 | 20.8% | 137 | 27.4% |
| | Protestants | 379 | 75.8% | 110 | 22.0% |
| | Muslims | 17 | 3.4% | 253 | 50.6% |
| **Occupation** | Formal sector workers | 31 | 6.2% | 40 | 8.0% |
| | Farmers (small/large scale) | 78 | 15.6% | 103 | 20.6% |
| | Self-employed (small/large business) | 162 | 32.4% | 190 | 38.0% |
| | Retired | 55 | 11.0% | 72 | 14.4% |
| | Unemployed | 174 | 34.8% | 95 | 19.0% |
| **Health insurance** | Insured | 337 | 67.4% | 302 | 60.4% |
| | Not insured | 163 | 32.6% | 198 | 39.6% |
| **Socioeconomic status^** | Lower SES (1–5) | 447 | 89.4% | 374 | 74.8% |
| | Higher SES (6–10) | 53 | 10.6% | 126 | 25.2% |
| **Family history of T2D** | History of T2D | 267 | 53.4% | 205 | 41.0% |
| | No history of T2D | 233 | 46.6% | 295 | 59.0% |
| **Time living with T2D** | <6 years | 191 | 38.2% | 166 | 33.2% |
| | ≥6 years | 309 | 61.8% | 334 | 66.8% |
| **Mean number of comorbidities [SD]** | | 500 | 3.4 [3.9] | 368 | 1.5 [0.8] |

Notes: Urban locations were Nairobi and Kiambu (Kenya) and Dar es Salaam (Tanzania); rural locations were Nyeri and Vihiga (Kenya) and Morogoro (Tanzania); ^ as measured by a self-report ladder scale where 1 represents low SES and 10 high SES.



For peer review only - http://bmjopen.bmj.com/site/about/guidelines.xhtml

**Table 2: Multivariate logistic regression results of potential predictors of COVID-19 vaccine uptake**

| Variable | Description | Kenya | | Tanzania | |
|---|---|---|---|---|---|
| | | AOR (p-value) | [95%CI] | AOR (p-value) | [95%CI] |
| **Place of residence** | Urban (ref) | 1.00 | | 1.00 | |
| | Rural | 1.09 (0.789) | [0.59-2.00] | 0.86 (0.605) | [0.49-1.50] |
| **Sex** | Female (ref) | 1.00 | | 1.00 | |
| | Male | 1.11 (0.696) | [0.65-1.92] | 1.30 (0.309) | [0.78-2.17] |
| **Marital status** | Not married (ref) | 1.00 | | 1.00 | |
| | Married | 1.56 (0.008) | [0.95-2.57] | 1.55 (0.086) | [0.94-2.57] |
| **Age group** | <40 years (ref) | 1.00 | | 1.00 | |
| | 40-49 years | 1.44 (0.402) | [0.61-3.37] | 1.21 (0.761) | [0.35-4.19] |
| | 50-59 years | 2.51 (0.029) | [1.10-5.72] | 1.56 (0.438) | [0.51-4.76] |
| | 60-69 years | 2.48 (0.038) | [1.05-5.84] | 2.11 (0.200) | [0.67-6.61] |
| | > 70 years | 2.46 (0.078) | [0.90-6.71] | 2.19 (0.270) | [0.54-8.82] |
| **Education level** | No education (ref) | 1.00 | | 1.00 | |
| | Primary education | 1.38 (0.596) | [0.42-4.57] | 1.47 (0.510) | [0.47-4.56] |
| | Secondary education | 1.54 (0.490) | [0.45-5.21] | 1.79 (0.345) | [0.53-6.03] |
| | Higher (college/university) education | 4.25 (0.049) | [1.00-18.03] | 4.07 (0.046) | [1.03-16.12] |
| **Religion** | Catholic (ref) | 1.00 | | 1.00 | |
| | Protestants | 0.87 (0.620) | [0.49-1.53] | 1.19 (0.565) | [0.66-2.13] |

For peer review only



For peer review only - http://bmjopen.bmj.com/site/about/guidelines.xhtml

| | | | | | |
|---|---|---|---|---|---|
| | Muslims | 0.67 (0.512) | [0.20-2.23) | 0.94 (0.822) | [0.55-1.62] |
| **Occupation status** | Formal sector workers (ref) | 1.00 | | 1.00 | |
| | Farmers | 2.86 (0.115) | [0.77-10.62] | 1.15 (0.777) | [0.44-3.00] |
| | Self-employed business | 1.33 (0.623) | [0.43-4.14] | 1.16 (0.742) | [0.47-2.87] |
| | Taking care of home | 1.05 (0.952) | [0.20-5.58] | | |
| | Retired | 1.26 (0.735) | [0.33-4.91] | 0.89 (0.809) | [0.34-2.32] |
| | Unemployed | 0.81 (0.728) | [0.26-2.58] | 0.99 (0.991) | [0.35-2.80] |
| **Health insurance** | Insured | 1.70 (0.026) | [1.07-2.70] | 1.81 (0.035) | [1.04-3.13] |
| | Not insured (ref) | 1.00 | | 1.00 | |
| **Socioeconomic status** | Lower SES (1–5) | 0.79 (0.522) | [0.38-1.63] | 0.62 (0.059) | [0.37-1.02] |
| **(ladder scale 1-10)** | Higher SES (6–10) (ref) | 1.00 | | 1.00 | |
| **Family history of T2D** | Yes | 1.42 (0.123) | [0.91-2.21] | 1.35 (0.189) | [0.86-2.10] |
| | No (reference) | 1.00 | | 1.00 | |
| **Time living with T2D** | <6 years | 0.75 (0.228) | [0.47-1.20] | 0.75 (0.249) | [0.46-1.22] |
| | ≥6 years (ref) | 1.00 | | 1.00 | |
| **Comorbidities** | Number of comorbidities | 1.08 (0.691) | [0.75-1.55] | 1.28 (0.042) | [1.01-1.61] |

Notes: AOR=Adjusted Odds Ratio; CI=Confidence Interval; ref=reference group

For peer review only

ISO 9001: 2015 certified
IFAKARA HEALTH INSTITUTE
research | training | services

African Population and Health Research Center

# Healthcare and Socio-economic Impacts of COVID-19 on Patients with type 2 Diabetes in Kenya & Tanzania

# Athari za Kiafya na Kiuchumi za UVIKO-19 kwa Wagonjwa wa Kisukari cha Aina ya 2 nchini Kenya na Tanzania

## Indepth Interview guide for individuals with type 2 diabets

## Mwongozo wa Mahojiano ya Kina kwa Wagonjwa wa  Kisukari cha Aina ya 2

Before we start the interview, I would like us to read the informed consent form together, and then you can let me know if you want to be interviewed.

Kabla hatujaanza mahojiano, ningependa tusome pamoja fomu ya ridha, kisha unaweza kunijulisha ikiwa unapenda kuhojiwa.

*Research assistant: (remember to collect the social demographic characteristics of the participants before starting the interview)*

Msaidizi wa utafiti: *(kumbuka kukusanya sifa za idadi ya watu kijamii ya washiriki kabla ya kuanza mahojiano)*

| **DEMOGRAPHIC INFORMATION**<br>**TAARIFA ZA KIDEMOGRAFIA** | |
|---|---|
| **Respondent name/code**<br>**Jina la mhojiwa/msimbo** | |
| **Age**<br>**Umri** | |
| **Education**<br>**Elimu** | |
| **Marital status**<br>**Hali ya ndoa** | |
| **Economic income/position**<br>**Mapato ya kiuchumi/nafasi** | |
| **Religion**<br>**Dini** | |

For peer review only

| | |
|---|---|
| **Occupation**<br>**Kazi** | |
| **Tribe**<br>**Kabila** | |
| **Village**<br>**Kijiji** | |
| **District**<br>**Wilaya** | |
| **Region**<br>**Mkoa** | |
| **Contact**<br>**Mawasiliano** | |

## Section A: Illness history
## Sehemu A: Historia ya Ugonjwa

**Please tell me more about your experience with diabetes condition since you were diagnosed with diabetes[FI *Instruction: Ask the respondent to think back to the time from when you were diagnosed]***

**Tafadhali niambie uzoefu wako kuhusiana na ugonjwa wa kisukari tangu ulipogunduliwa na ugonjwa wa kisukari** *[Maelekezo ya FI: Mwambie mhojiwa afikirie kipindi tangu alipogundua kuwa na ugonjwa wa kisukari]*

**Dodosa:**

a) What symptoms,if any,did you experience?
Je, ulikuwa na dalili gani?

b) How did you feel about being diagnosed with diabetes?
Ulijisikiaje ulipogundulika kuwa na ugonjwa wa kisukari?

c) How did your family, friends and community members respond to you having diabetes?
Je familia yako,marafiki na wanajamii waliopokea vipi suala la ugonjwa wako wa kisukari?

1. **Do you have any other health conditions apart from diabetes?**
**Je, una shida nyingine ya kiafya mbali na ugonjwa wa kisukari?**
**If the response is yes, probe the following**
**Ikiwa jibu ni ndiyo, dodosa yafuatayo:**

a) Please briefly talk about your experience living with diabetes ?
Tafadhali nieleze kwa ufupi ni jinsi gani umekuwa ukiishi na ugonjwa huu wa kisukari?

For peer review only

b) Has it been easy or difficult for you to live with all these health problems? explain more
Je imekuwa rahisi au ngumu kwako kuishi na shida zote hizi za kiafya? eleza zaidi

c) Has this condition affected how you control your diabetes?
Je, hali hii imeathiri jinsi unavyodhibiti hali yako ya ugonjwa wa kisukari ?

## Section B: Patient experience in illness management before COVID-19

## Sehemu B: Uzoefu wa mgonjwa katika udhibiti wa magonjwa kabla ya UVIKO-19

In this section we are going to discuss questions about how you have **MANAGE** your diabetes and access to diabetes care services **BEFORE** the COVID-19 period, that is before March 2020

Katika sehemu hii tutajadili maswali kuhusu namna ambavyo umeweza **KUDHIBITI** ugonjwa wako wa kisukari na upatikanaji wa huduma za matibabu ya kisukari **KABLA** ya kipindi cha UVIKO-19, yani kabla ya Machi 2020

2. **There are different ways of managing diabetes, please tell me how you were managing your diabetes in the period before COVID-19 (*do not forget asking about modern medical ways such as taking medication, injections, exercise, diet and using natural remedies*)**

   **Kuna njia mbalimbali za kudhibiti ugonjwa wa kisukari, tafadhali niambie jinsi ulivyokuwa ukidhibiti ugonjwa wako wa kisukari katika kipindi cha kabla ya UVIKO-19** *(usiache kudadisi kuhusu njia za kitabibu za kisasa kama kunywa dawa, sindano, mazoezi, chakula na kutumia njia za asili)*

3. **Thinking about how you have been managing diabetes** (*provide an example of the activity that the patient mentioned above)* **how has your management of diabetes changed over time (here we are still referring to the period before COVID-19)?**

   **Fikiria namna ambavyo umekuwa ukidhibiti ugonjwa wa kisukari***(toa mfano wa shughuli ambayo mgonjwa alitaja hapo juu)* **je udhibiti wako wa ugongwa wa kisukari umekuwa ukibadilika? (hapa bado tunazungumzia kipindi cha kabla ya UVIKO-19)?**

   Probe: What has changed? Please tell us more
   Dodosa: Nini kimebadilika? Tafadhali tuelezea zaidi.

4. **What type of assistance have you been receiving in managing your diabetes? (*remind the participant that you are reffering to the period before COVID-19*)**

**Umekuwa ukipokea msaada wa aina gani katika kudhibiti ugonjwa wako wa kisukari?** (mkumbushe mshiriki kuwa unarejea kipindi cha kabla ya UVIKO-19)

Probe: Did anyone help you to take care of your diabetes? tell us more

Dodosa: Je, kuna mtu yeyote aliyekusaidia kukuhudumia katika ugonjwa wako kisukari?

Tell us more:Family member, health care provider, traditional healers?

Tuambie Zaidi (uliza kuhusu: mwanafamilia, mhudumu wa afya, waganga wa kienyeji?)

Probe: Was the assistance helpful? why?

Dodosa: Je, msaada huo ulisaidia? kwa nini?

We have spoken about how you manage your diabetes. Now lets talk about how you **ACCESS** diabetes health care services in the period **BEFORE** COVID-19.

Tumezungumza juu ya namna unavyo **DHIBITI** ugonjwa wako wa kisukari. Sasa hebu tuzungumze kuhusu **UFIKIAJI** wako wa huduma za matibabu ya ugonjwa wa kisukari katika kipindi cha kabla ya UVIKO-19

5. **Tell us about where you sought diabetes health care services?**
   **Tuambie unapata wapi huduma za matibabu ya ugonjwa wa kisukari?**

**Ddosa:**

a) Probe: what type of services did you receive from the health facility?
   Je ni aina gani ya huduma ulizopokea kutoka kwa kituo cha afya?
b) Probe: how helpful were the services that you received? Tell us more
   Je huduma ulizopokea zilikua na manufaa kiasi gani? Tuambie Zaidi
c) Probe: how did the services met your expectations? Why?
   Je ni kwa namna gani huduma za kiafya zilikidhi vipi matarajio yako? Kwa nini?

6. **What information did you get about controlling your diabetes?**
   **Dodosa: Ni taarifa zipi umepata juu ya kudhibiti ugonjwa wako wa kisukari?**

a) Probe: who provided that information?
   Nani alikupa taarifa hizo?
b) Probe: was the information helpful? how?
   Je taarifa hizi zilikuwa na msaada? Ki vipi?

*Think about things that might have made accessing diabetes care services easy or difficult in the period before COVID-19 (**before March** 2020)*

*Fikiri kuhusu mambo ambayo huenda yalifanya upatikanaji wa huduma za matibabu ya kisukari kuwa rahisi au ngumu katika kipindi cha kabla ya UVIKO-19 (**kabla ya Machi** 2020)*

For peer review only

7. **How difficult/easy was it for you to access diabetes services in the period before COVID-19? (why)**
**Je, ilikuwa vigumu/rahisi vipi kwako kupata huduma za matibabu ya ugonjwa wa kisukari kabla ya UVIKO-19? (kwanini?)**

**Dodosa:**

a) Probe: what was the difficult/easy aspect?
Ni kitu gani hasa kilikuwa gumu/rahisi kwako?

b) Probe: how did you handle this difficult?
Je ni kwa namna gani uliweza kukabiliana na ugumu huo?

c) Was it easy for you to handle this difficult?
Je ilikuwa rahisi kwako kutatua hiyo hali ngumu/changamoto hiyo?
d) How did that challenge / situation affect your diabetes management?
Je chagamoto/hali hiyo ngumu iliathiri vipi kusimamizi wako wa ugonjwa wa kisukari?

e) Probe: did you find help in managing this diffiuclt? Tell us more
Je, ulipata msaada katika kudhibiti changamoto hiyo/hali hiyo ngumu? Tueleze Zaidi

**Ask: Can you tell me exactly before COVID-19 you were experiencing any difficulty in the following aspects?**

**Uliza: Unaweza kuniambia haswa kabla ya UVIKO-19 ulikuwa unapata ugumu wowote katika vipengele vifuatavyo?**

- Transport, cost, availability of the services – how?
- Usafiri, gharama za kwenda kwenye huduma za afya?
- Access to medication- how?
  Upatikanaji wa huduma - kivipi?
- Access to medicine - how?
  Upatikanaji wa dawa - vipi?
- See a doctor - How?
  Kumuona daktari-Kivipi?
- Adhering medical advice? - how?
- Kuzingatia ushauri wa matibabu? - kivipi?
- Distress, fear? What exactly were you thining ?
  Msongo wa mawazo, hofu? Ulikuwa unawaza nini hasa?
- Food? (how)
  Chakula? Kivipi?
- Getting councelling, how?
  Kupata ushauri, kivipi?
- Support from relatives,how?
  Msaada kutoka kwa ndugu, kivipi?

For peer review only

## DURING COVID-19 PERIOD
## WAKATI WA UVIKO-19

### Section C: Patient experience in illness management since the beginning of the COVID-19 pandemic
### Sehemu C: Uzoefu wa mgonjwa katika udhibiti wa ugonjwa tangu mwanzo wa janga la UVIKO-19

In this section we are going to talk about how you have **MANAGED** your diabetes, and how you have **ACCESSED** the diabetes care services **SINCE** COVID-19 (We mean the period after March 2020)

Katika sehemu hii tutazungumzia namna gani ambavyo ulikuwa **UKIDHIBITI** ugonjwa wako wa kisukari, na jinsi ulivyofikia huduma za **UDHIBITI** wa ugonjwa wa kisukari **TANGU** UVIKO-19 kuanza (tunamaanisha kipindi baada ya Machi 2020)

8. **Please tell me what ways you have been using to control your diabetes during COVID-19? (After March 2020)**
   **Tafadhali niambie njia gani ulikuwa ukitumia kudhibiti ugonjwa wako wa kisukari wakati wa UVIKO-19? (Baada ya Machi 2020)**

   ***Probe: Did these ways of controlling diabetes helped you in COVID-19 period? If not, why? If helped, why do you think so?***
   ***Probe:Probe:*** *Je njia hizi za udhibiti ugonjwa wa kisukari zimekusaidia wakati huu wa UVIKO-19? Kama hazikusaidia unafikiri kwa nini? Kama zilisaidia unafikiri kwa nini?*

9. **Are there any changes in how you were managing diabetes before COVID-19 and during COVID-19? (*give an example of what respondent mentioned above like; using medicine, nutrition, exercise, etc ask if it changed)***
   **Je kuna mabadiliko yoyote katika kudhibiti ugonjwa wa Kisukari kabla ya UVIKO-19 na wakati wa UVIKO-19?** *(toa mfano, jambo ambalo mgonjwa alitaja hapo juu kama vile: kumeza vidonge, lishe, mazoezi, uulize kama kilibadilika)*

   **Probe: What has changed? Please tell me more**
   **Dodosa:** Nini kimebadilika? Tafadhali nielezee Zaidi

   **Probe: What do you think has contributed to these changes?**
   **Dodosa:** Unafikiri nini kimechangia mabadiliko haya?

   **Probe: Did you receive any support to help you to control these changes? what help, if any?**

**Dodosa:** Je ulipata msaada wowote wa kukusaidia kudhibiti mabadiliko haya? msaada gani?kama upo

**10. Has your health related to diabetes changed since COVID pandemic started?**

**Je, afya yako kuhusiana na ugonjwa wa kisukari imebadilika tangu kuanza kwa janga la UVIKO-19?**

**Probe: What has specifically changed?**

**Dodosa:** Nini hasa kimebadilika?

**Uliza:**

- Changes in blood glucose level
  Mabadiliko katika kiwango cha sukari kwenye damu
- Emerged health problems such as eye problems, wounds that do not heal well, feeling irritated in the legs or feet.
- Matatizo mapya mfano matatizo ya macho, vidonda ambavyo haviponi vizuri, hisia za kuwashwa kwenye miguu au nyayo.
  -Adhere to your medicine (how?)
- Kuzingatia utaritbu wa kutumia dawa zako (vipi?)

-Fear about diabetes illness (if so, why/) what were you afraid of
Hofu kuhusu ugonjwa wa kisukari (ikiwa ni hivyo, kwa nini?) ulikuwa unahofia nini?

- Depression? (If yes,how depression has affected the way you were and how you are coping with your Diabete)
  Msongo wa mawazo? (Ni jinsi gani msongo wa mawazo umeadhiri namna ambavyo ulivyokuwa na unakabiliana na ugonjwa wako wa Kisukari)

**Dodosa:**

a) Probe: What do you think has contributed to these changes?
   Unadhani nini kisababisha mabadiliko haya?

b) Probe: how have you been handling these changes? has it been easy/difficult?
   Umekuwa ukishughulikiaje mabadiliko haya? imekuwa rahisi/vigumu?

   Probe: have you been worried about these changes? why?
   Dodosa: je umekuwa na wasiwasi kuhusu mabadiliko haya? kwa nini?

*Think about the things that may have made you to access to diabetes treatment easier or harder during COVID-19 (after March 2020)*



For peer review only

*Fikiri kuhusu mambo ambayo huenda yalifanya kupata huduma za matibabu ya kisukari kuwa rahisi au ngumu wakati wa UVIKO-19 (baada ya Machi 2020)*

**11. Was it difficult/easy was it for you to control diabetes during COVID-19? (After March 2020)**

**Je ilikuwa ngumu/rahisi vipi kwako kudhibiti ugonjwa wa kisukari katika kipindi cha UVIKO-19? (Baada ya Machi 2020)**

Probe: What exactly was difficult for you to control in your diabete? Explain

**Dodosa:** Ni nini hasa kilikuwa kigumu kwako katika kudhibiti ugonjwa wako wa kisukari? Eleza

Probe: How did you manage to deal with this difficult situation? Was it easy for you to handle this difficulty?

**Dodosa:** je umewezaje kukabiliana na hali hii ngumu? Je imekuwa rahisi kwako kushughulikia ugumu huu? Je, ulipata msaada katika kudhibiti hali hii ngumu?

Probe: Did you get help in managing this difficult situation?Explain more

**Dodosa:** je ulipata msaada katika kudhibiti ugonjwa wako wa kisukari? Tuelezee Zaidi

Ask:can you tell me if you experienced any of the following problems?

Uliza: unaweza kuniambia kama ulipata matatizo yanayohusiana na haya yafuatayo?

- Transport, costs, access to health care - how?
  Usafiri, gharama, upatikanaji wa huduma za afya - kivipi?
- Access to medicine - how?
  Upatikanaji wa dawa - vipi?
- Adhere to medica advise, how?
- Kuzingatia ushauri wa matibabu? - vipi?
- Depression? What exactly were you thinking?
  Msongo wa mawazo? Ulikuwa unawaza nini hasa?
- Fear? what exactly were you afraid of?
  Hofu? ulikuwa unahofia nini hasa?

12. **What support have you been getting in managing your diabetes? (*Remind participant that you are referring to the period AFTER MARCH 2020, during COVID-19* )**

**Je umekuwa ukipata msaada gani katika kudhibiti ugonjwa wako wa kisukari?***(mkumbushe mshiriki kuwa unarejea kipindi cha **BAADA YA MACHI 2020,** wakati wa UVIKO-19)*

Probe: Did anyone help you in controlling your diabetes during COVID-19?

For peer review only

Dodosa: Je, kuna mtu yeyote aliyekupa msaada katika kudhibiti kisukari chako wakati wa UVIKO-19?

**Ask**: family member, health care provider, traditional healer).Tell us more
**Uliza:**mwanafamilia, mhudumu wa afya, waganga wa kienyeji).Tulezee zaidi

Probe: Did that support help? how that support helped you?
Dodosa**:** Je, msaada huo ulikusaidia? Ulikusaidiaje?

13. In your opinion, what can help diabete patients to manage their illness effectively during the COVID-19 epidemic? [Instructions: You can refer to the diabetes control measure/ways the respondent mentioned earlier]

**Kwa maoni yako ni kitu gani kinaweza kuwasaidia wagonjwa wa kisukari kudhibiti ugonjwa wao ipasavyo wakati wa janga la UVIKO-19?** [Maagizo: Unaweza kurejea njia za udhibiti wa kisukari mhojiwa alitaja hapo awali]

We have talked about how to control your diabetes. Now let's talk about **the AVAILABILITY** of diabetic health care that you may have accessed in the pre-COVID-19 period

Tumezungumza juu ya jinsi ya kudhibiti ugonjwa wako wa kisukari. Sasa hebu tuzungumze kuhusu **UPATIKANAJI** wa huduma za afya za kisukari ambazo unaweza kuwa umefikia katika kipindi cha kabla ya UVIKO-19

**14. Was it difficult / easy for you to get diabete health services during COVID-19? (after March 2020)(why?)**
**Je, ilikuwa vigumu/rahisi kwako kupata huduma za afya za ugonjwa wa kisukari katika kipindi cha UVIKO-19? (baada ya Machi 2020) (kwanini?)**

Probe: What exactly was the difficulty in getting diabetes care during COVID-19? How?
Dodosa: ni jambo gani lilikuwa kigumu katika kupatikanaji wa huduma za ugonjwa wa kisukari wakati wa UVIKO-19? Ki Vipi?

Probe: How did you manage to deal with this difficult situation? Was it easy for you to handle this difficulty? Have you found help in managing this difficult situation?
Dodosa: umewezaje kukabiliana na hali hii ngumu? Je ilikuwa rahisi kwako kushughulikia ugumu huu? Je, umepata usaidizi katika udhibiti wa hali hii ngumu?

For peer review only

Probe:Did you get help getting diabetes services during this COVID-19? Tell us more
Dodosa: je umekuwa ukipata msaada katika kupata huduma za matibau ya ugonjwa wa kisukari wakati wa huu wa UVIKO-19? Tuelezee zaidi

*Think about the things that may have contributed to access diabetes services easier or difficulty during COVID-19 (after March 2020)*

*Fikiria kuhusu mambo ambayo huenda yamesababisha kufikia huduma za ugonjwa wa kisukari kuwa rahisi au ngumu katika kipindi cha UVIKO-19 (baada ya Machi 2020)*

Ask: Can you tell me exactly if you are experiencing any challenges in accessing diabetes services during COVID-19?
Uliza: Je unaweza kuniambia haswa iwapo unakutana na changamoto zozote katika kupata huduma za ugonjwa wa kisukari wakati wa UVIKO-19?
Probe the following challenges are below:
Dodosa changamoto zifuatazo hapa chini:

- Transport, costs, access to services - how?
  Usafiri, gharama, upatikanaji wa huduma - vipi?
- Access to medicine - how?
- Upatikanaji wa dawa - vipi?
- Adhering to medical advice? - how?
- Kuzingatia ushauri wa matibabu? - vipi?
- Family support
  Msaada wa familia
- Fear / stress, how
  Hofu/ dhiki, vipi?

**15. What medical suport did you receive in managing/controlling your diabetes during COVID-19?**
**Je umepokea msaada gani wa matibabu ya kiafya ulioyopata katika kudhibiti ugonjwa wako wa kisukari wakati wa UVIKO-19?**

Probe: Where did you get diabetes care during COVID-19?
Dodosa: Ulipata wapi huduma ya ugonjwa wa kisukari wakati wa UVIKO-19?
Probe: How useful were the services you received in managing diabetes during COVID-19? Tell me more
Dodosa: Je, huduma ulizopokea katika kudhibiti ugonjwa wa kisukari wakati wa UVIKO zilikufaa kiasi gani? Niambie Zaidi

Probe: Did you receive any additional services to help control/manage diabetes during COVID-19 as opposed to starting BEFORE COVID-19? Is this important? Why?

For peer review only

Dodosa: Je ulipata huduma zozote za ziada kukusaidia udhibiti ugonjwa wa kisukari wakati wa UVIKO-19 tofauti na mwanzo KABLA ya UVIKO-19? Je hili ni la muhimu? Kwa nini?

Probe: Has health care been able to help diebetes patients access their services during this COVID-19 period? If so why not? if not why not
Dodosa: Je huduma za afya zimeweza kuwasaidia wenye ugonjwa wa kisukari kupata huduma zao wakati huu wa UVIKO-19? Kama ndiyo ni kwanini? kama sio ni kwa nini?

**16. What do you think should be done to help diabete patients to better manage their illness during health events like COVID-19? what exactly should be done?**
**Je nini unafikiri kifanyike ili kuwasaidia wagonjwa wenye kisukari kudhibiti vizuri ugonjwa wao wakati wa majanga ya kiafya kama UVIKO-19? nini hasa kifanyike?**

## Section D: Health information and Knowlege about COVID-19 pandemic and Practices

## Sehemu D: Taarifa za afya,Maarifa na Uzoefu/Mbinu kuhusu janga la UKIVO-19

### Knowledge about COVID-19

### Ufakamu kuhusu UVIKO-19

**18.Can you tell me about your reaction when you heard about COVID-19?**
**Je, unaweza kuniambia ulijisikiaje uliposikia kuhusu UVIKO- 19?**

**19.Where did you hear about UVIKO-19?**
**Ulisikia wapi kuhusu UVIKO-19?**
**20.How is COVID-19 transmitted?**
**Je UVIKO-19 inaambukizwa vipi?**
**21.How COVID-19 is prevented?**
**Je UVIKO-19 inaepukwa vipi?**
**22. What have you personally been doing to avoid getting COVID-19?**
**Probes**
**Je wewe mwenyewe umekuwa ukifanya nini ili kuepuka UVIKO-19?**
**Probes**

- **How easy / difficult has it been for you to wear masks?**
  Je imekuwa rahisi/vigumu vipi kwako kuvaa barakoa?
- Has it been easy / hard for you to clean yourself?
  imekuwa rahisi/ngumu kwako kujitakasa?
- How easy / difficult has it been for you to keep social distance?
  Je imekuwa rahisi/vigumu vipi kwako kukaa kwa umbali katika kijamii?
- Did you use any natural remedies to protect yourself from COVID-19?
  Je, ulitumia dawa zozote za asili kujikinga na UVIKO-19?
- If so, ask what were those remedies ?
  Ikiwa ndiyo, uliza dawa hizi zilikuwa ni zipi?

For peer review only

- To what extent the natural remedies helped?
- Je, kwa kiasi gani dawa hizi za asili zinasaidia ?
- were these drugs available?
- Je dawa hizi zilipatikana?
- How did you know these natural remedies?
- Je ulizijuaje dawa hizi?

If not, ask why respondent did not use these traditional remedies?
Ikiwa hapana, uliza kwa nini haukutumia dawa hizi za kienyeji?

**23. Do you think COVID-19 can affect your diabetes? if so, explain to me a little bit?**
**Je UVIKO-19 inaweza kuathiri ugonjwa wako wa kisukari? kama ndiyo nieleze kidogo?**

## Information about COVID-19
## Taarifa kuhusu UVIKO-19

**24. Please tell me more about any information you have heard or received about your diabetes management since the COVID-19 epidemic began**

**Tafadhali niambie zaidi kuhusu taarifa yoyote uliyosikia au kupokea kuhusu udhibiti wa ugonjwa wako wa kisukari tangu janga la UVIKO-19 lianze?**

**Probes: a**

**a.** Were you specifically informed about how people with diabetes should manage their illness to protect themselves from COVID-19?

**Dodosa:**Je, ulipewa taarifa namna ambavyo watu wenye ugonjwa wa kisukari wanapaswa kudhibiti ugonjwa wao ili kujikinga na UVIKO-19?

What did the information mean?
Je, taarifa hizo zilihusu nini?
Where did you get the information?
Umepata wapi taarifa?

**b.** How helpful were the information you received in controlling your diabetes?
Je, taarifa ulizopokea zilikuwa na manufaa kiasi gani kwako katika kudhibiti ugonjwa wako wa kisukari?

**c.** Did you know about where you can ask questions or get advice for your diabetes control or any other health condition you had during this COVID-19?
Je, ulikuwa unafahamu kuhusu ni wapi unapoweza kuuliza maswali au kupata ushauri kwa ajili ya udhibiti ungonjwa wako wa kisukari au hali yoyote yakiafya uliyokuwa nayo wakati wa huu UVIKO-19?

## Impacts of COVID -19 and diabetes
**Athari za UVIKO-19 na ugonjwa wa kisukari**

For peer review only

**In this section we aim to better understand your views on the effects of the COVID-19 pandemic and diabetes**

**Katika sehemu hii tuna lengo la kuelewa zaidi maoni yako kuhusu athari za janga la UVIKO-19 na ugonjwa wa kisukari**

**25. Since COVID-19 started, have you had any other health problems?**
**Tangu UVIKO-19 ilipoanza, je, umepata mtatizo mengine ya kiafya?**

**Probe:**

a. If so, please explain briefly about these health problems
Ikiwa ndiyo, tafadhali elezea kwa ufupi kuhusu matatizo hayo ya kiafya

b. Ask about other problems such as:
Uliza kuhusu matatizo mengine kama vile:
- stress, lack of sleep, inability to interact with people others, fear of COVID or death, mental stress
- mfadhaiko, ukosefu wa usingizi, uwezo wa kushirikiana na watu wengine, hofu itokanayo na UVIKO au kifo, msongo wa mawazo
- concerns about household issues eg access to food, electricity, water, family care
  wasiwasi wa maswala ya nyumbani mfano kuhusu upatikanaji wa chakula, umeme, maji, utunzaji wa familia
- income (employment /income-generating activities)any other?
- mapato (kazi/ajira; shughuli za kujiongezea kipato)
- nyingine yoyote?

c. Ask: what respondent think cause that situation?
Muulize: Ni kitu gani anafikiri kinaweza kuwa kilisababisha hali kama hizo

d. Ask how the respondent dealt with that situation and how?
Uliza ikiwa aliweza kukabiliana na hali hizo na jinsi gani?

e. Ask if these conditions have affected the patient's control over diabetes
Uliza kama hali hizi zimeathiri jinsi mgonjwa alivyodhibiti ugonjwa wa kisukari

f. Ask if these conditions have affected the diabetes treatment services provided
Uliza iwapo hali hizi zimeathiri huduma za matibabu ya ugonjwa wa kisukari iliyotolewa?

**26. How has diabetes affected your COVID-19 management?**
**Je, kwa namna gani ugonjwa wa kisukari umeathiri usimamizi/udhibiti wako kwenye UVIKO-19?**

For peer review only

**Section F: Opinions about COVID-19 Vaccine**
**Sehemu F: Maoni kuhusu Chanjo ya UVIKO-19**
*I would also like to know your views on the COVID-19 vaccine*
*Ningependa pia kujua maoni yako kuhusu chanjo ya UVIKO-19*

**27. What is your opinion on the COVID-19 vaccine?**
**- Je, una maoni gani kuhusu chanjo ya COVID-19?**
Probe: is vaccine good or bad and why?
Dodosa: je chanjo ni nzuri au mbaya na kwanini?

**28. Have you received the COVID-19 vaccine?**
**Je, umepata chanjo ya UVIKO-19?**
If so, what is it that has motivated you?
Kama ndiyo, ni kitu gani kimekuhamasisha?
If not, what are some of the things that keep you from getting vaccinated?
Ikiwa hapana, ni mambo gani yanayozuia usikubali kupata chanjo?

**29. Is there anything you would like to ask or talk about about diabetes that we have not discussed?**

**Je, kuna kitu chochote ambacho ungependa kuuliza au kuzungumzia kuhusu ugonjwa wa kisukari ambacho hatujajadili?**

***Reseacher: Thanks the participant for his /her valuable time and asks if they have any questions***

***Mtafiti: Anashukuru mshiriki kwa muda wake wa thamani na kuuliza kama wana swali lolote***

**...................................END/ MWISHO..............................................**

For peer review only

# Appendix

**Appendix Table 1: Multivariate Modified Poisson regression results of potential predictors of COVID-19 vaccine uptake**

| Variable | Description | Kenya | | Tanzania | |
|---|---|---|---|---|---|
| | | RR (p-value) | (95%CI) | RR (p-value) | (95%CI) |
| **Place of residence** | Urban (ref) | 1.00 | | 1.00 | |
| | Rural | 1.01 (0.900) | (0.90-1.12) | 0.90 (0.554) | (0.63-1.29) |
| **Sex** | Female (ref) | 1.00 | | 1.00 | |
| | Male | 1.02 (0.751) | (0.91-1.14) | 1.17 (0.355) | (0.84-1.63) |
| **Marital status** | Not married (ref) | 1.00 | | 1.00 | |
| | Married | 1.10 (0.118) | (0.98-1.25) | 1.37 (0.085) | (0.96-1.97) |
| **Age group** | <40 years (ref) | 1.00 | | 1.00 | |
| | 40-49 years | 1.15 (0.411) | (0.82-1.62) | 1.16 (0.760) | (0.45-3.00) |
| | 50-59 years | **1.33 (0.071)** | **(0.98-1.82)** | 1.38 (0.463) | (0.59-3.24) |
| | 60-69 years | **1.33 (0.071)** | **(0.98-1.83)** | 1.72 (0.214) | (0.73-4.03) |
| | > 70 years | **1.33 (0.085)** | **(0.96-1.85)** | 1.74 (0.249) | (0.68-4.44) |
| **Education level** | No education (ref) | 1.00 | | 1.00 | |
| | Primary education | 1.07 (0.690) | (0.78-1.46) | 1.41 (0.481) | (0.55-3.62) |
| | Secondary education | 1.09 (0.583) | (0 80-1.50) | 1.63 (0.327) | (0.61-4.36) |
| | Higher education | 1.27 (0.144) | (0.92-1.77) | **2.63 (0.065)** | **(0.94-7.35)** |

For peer review

| | | | | | |
|---|---|---|---|---|---|
| **Religion** | Catholic (ref) | 1.00 | | 1.00 | |
| | Protestants | 0.97 (0.611) | (0.87-1.09) | 1.11 (0.581) | (0.76-1.61) |
| | Muslims | 0.87 (0.448) | (0.61-1.25) | 0.95 (0.770) | (0.67-1.35) |
| **Occupation status** | Formal workers (ref) | 1.00 | | 1.00 | |
| | Farmers | 1.18 (0.137) | (0.95-1.46) | 1.08 (0.809) | (0.57-2.07) |
| | Self-employed business | 1.05 (0.631) | (0.87-1.26) | 1.10 (0.765) | (0.58-2.08) |
| | Taking care of home | 0.98 (0.900) | (0.69-1.39) | | |
| | Retired | 1.03 (0.825) | (0.82-1.28) | 0.93 (0.819) | (0.49-1.75) |
| | Unemployed | 0.91 (0.384) | (0.73-1.13) | 0.98 (0.949) | (0.46-2.07) |
| **Health insurance** | Insured | 1.14 (0.035) | (1.01-1.29) | 1.56 (0.045) | (1.01-2.41) |
| | Not insured (ref) | 1.00 | | 1.00 | |
| **Socioeconomic status (ladder scale 1-10)** | Lower SES (1–5) | 0.96 (0.637) | (0.82-1.13) | 0.74 (0.075) | (0.53-1.03) |
| | Higher SES (6–10) (ref) | 1.00 | | 1.00 | |
| **Family history of T2D** | Yes | 1.08 (0.130) | (0.98-1.19) | 1.22 (0.189) | (0.91-1.65) |
| | No (reference) | 1.00 | | 1.00 | |
| **Time living with T2D** | <6 years | 0.93 (0.208) | (0.84-1.04) | 0.82 (0.266) | (0.57-1.17) |
| | ≥6 years (ref) | 1.00 | | 1.00 | |
| **Comorbidities** | Number of comorbidities | 1.02 (0.629) | (0.95-1.10) | 1.18 (0.022) | (1.02-1.35) |

For peer review only
